# Integration of untargeted metabolomics with transcriptomics reveals active metabolic pathways

**DOI:** 10.1007/s11306-014-0713-3

**Published:** 2014-08-03

**Authors:** Kyuil Cho, Bradley S. Evans, B. McKay Wood, Ritesh Kumar, Tobias J. Erb, Benjamin P. Warlick, John A. Gerlt, Jonathan V. Sweedler

**Affiliations:** 1 Institute for Genomic Biology, University of Illinois at Urbana-Champaign, Urbana, IL 61801 USA; 2 Department of Chemistry, University of Illinois at Urbana-Champaign, 600 S. Mathews Ave., Urbana, IL 61801 USA; 3 Institute for Microbiology, Swiss Federal Institute of Technology (ETH) Zurich, CH-8093 Zurich, Switzerland; 4 Department of Biochemistry, University of Illinois at Urbana-Champaign, Urbana, IL 61801 USA

**Keywords:** Active pathway detection, Isoprenoid biosynthesis, Liquid chromatography–mass spectrometry, Metabolomics, Methionine salvage, Quantitative real time polymerase chain reaction, Transcriptomics

## Abstract

While recent advances in metabolomic measurement technologies have been dramatic, extracting biological insight from complex metabolite profiles remains a challenge. We present an analytical strategy that uses data obtained from high resolution liquid chromatography–mass spectrometry and a bioinformatics toolset for detecting actively changing metabolic pathways upon external perturbation. We begin with untargeted metabolite profiling to nominate altered metabolites and identify pathway candidates, followed by validation of those pathways with transcriptomics. Using the model organisms *Rhodospirillum rubrum* and *Bacillus subtilis*, our results reveal metabolic pathways that are interconnected with methionine salvage. The rubrum-type methionine salvage pathway is interconnected with the active methyl cycle in which re-methylation, a key reaction for recycling methionine from homocysteine, is unexpectedly suppressed; instead, homocysteine is catabolized by the *trans*-sulfuration pathway. Notably, the non-mevalonate pathway is repressed, whereas the rubrum-type methionine salvage pathway contributes to isoprenoid biosynthesis upon 5′-methylthioadenosine feeding. In this process, glutathione functions as a coenzyme in vivo when 1-methylthio-d-xylulose 5-phosphate (MTXu 5-P) methylsulfurylase catalyzes dethiomethylation of MTXu 5-P. These results clearly show that our analytical approach enables unexpected metabolic pathways to be uncovered.

**Electronic supplementary material:**

The online version of this article (doi:10.1007/s11306-014-0713-3) contains supplementary material, which is available to authorized users.

## Introduction

We have developed a systems biology approach that combines an automated liquid chromatography (LC)–mass spectrometry (MS)-based metabolomics pipeline (Raamsdonk et al. 2001) with transcriptomics and use it to construct a holistic picture of the methionine salvage metabolism in several model organisms. The methionine salvage pathway is universal and present in many organisms, from unicellular bacteria to plants and animals, with some variations (Albers 2009; Wray and Abeles 1995; Trackman and Abeles 1983). Two alternative methionine salvage pathways are known that convert 5′-methylthioadenosine (MTA), a by-product of *S*-adenosyl-l-methionine (SAM)-dependent polyamine biosynthesis, to methionine. The classical pathway, as determined in *Bacillus subtilis* (*B.* *subtilis*), is an eight-step pathway in which the carbon and sulfur skeleton of methionine is completely synthesized from the methylthioribose moiety of MTA (Ashida et al. 2003). The second pathway, recently reported by our group (Erb et al. 2012), was discovered in *Rhodospirillum rubrum* (*R.* *rubrum*) and involves a bifurcation of the methylthioribose molecule between methionine and isoprenoid biosynthesis. In *R.* *rubrum*, the pathway intermediate, methylthioxylulose-5-phosphate, is cleaved into methanethiol and the non-mevalonate isoprenoid precursor, 1-deoxy-d-xylulose 5-phosphate (DXP), in a novel enzymatic reaction that is dependent on 1-methylthio-d-xylulose 5-phosphate (MTXu 5-P) methylsulfurylase (Warlick et al. 2012). Whereas methanethiol can be further converted to methionine by the action of an *O*-acetyl-l-homoserine sulfhydrylase, the remaining carbon skeleton of MTA is channeled into isoprenoid biosynthesis.

In recent years, using metabolite profiling as a means to reveal novel aspects of cellular metabolism has gained popularity because it provides insight into complex regulatory processes as well as direct functional information on metabolic phenotypes (Fiehn et al. 2000; Zhao et al. 2013). Integration of metabolite profiles with transcriptomics data (Kresnowati et al. 2006; Bradley et al. 2009; Lei et al. 2011) or metabolic networks (Cakir et al. 2006) has advanced our understanding of biological systems to the next level. LC coupled to high resolution, accurate mass spectrometric platforms enables the detection of many thousands of features, but requires high throughput data handling methods to convert the raw data into biological discovery (Wei et al. 2011; Eliasson et al. 2012).

Multivariate statistical methods are routinely used to analyze complex data to discover biological patterns (Fernie et al. 2004). However, converting this wealth of information into an understanding of biological function remains challenging. Even though pathway-level analysis has been applied to gene expression data (Tavazoie et al. 1999; Curtis et al. 2005), only recently have tools for analyzing metabolite profiles in the context of predefined biological metabolite sets been reported (Xia and Wishart 2010, 2011; Chagoyen and Pazos 2011; Kankainen et al. 2011). These approaches have been predominately applied to study human and mammalian metabolomics because many biological sets and their constituent metabolites are well defined for these species (Sreekumar et al. 2009; Deo et al. 2010; Putluri et al. 2011a, b). However, the application of these approaches to pathway discovery in bacteria is hampered by the following issues: (i) there is no reference metabolome that can be measured by a specific analytical platform; (ii) time-consuming data pre-processing is required to reduce false positives and false negatives in the peak detection process; (iii) detected peaks should be annotated in advance with high confidence before applying metabolite set enrichment analysis (MSEA), a counterpart of gene set enrichment analysis (GSEA) (Mootha et al. 2003; Subramanian et al. 2005); and (iv) it is not always obvious which metabolite sets (i.e., pathways) should be tested, especially when a goal is to discover the unexpected pathways in vivo.

Here, we integrated untargeted metabolomics with transcriptomics [quantitative real time polymerase chain reaction (qRT-PCR) and RNA sequencing (RNAseq)] to correlate specific changes in bacterial growth conditions and a genetic knockout with changes in the activities of metabolic pathways. We used the concept of seed metabolites, which we define as the metabolites showing higher abundance changes upon perturbation, unambiguous formula determination and search hits in a database. These seed metabolites and a dynamic build-up of metabolite sets were used for the detection of actively changing metabolic pathways observed in the raw LC–MS data. In this process, we used the refined mass spectral features from the LC–MS data, regardless of the metabolite annotations, as a reference metabolome for our enrichment analysis, which is based on the same procedure used in GSEA. Putative metabolite annotation of the seed metabolites was carried out with high confidence and high coverage, and with a low number of false positives. Metabolite annotation was followed by a dynamic build-up of metabolite sets based on the pathway information extracted from the seed metabolites. Detected active pathways were then validated with transcriptomics. We applied this analytical strategy to unravel metabolic pathways linked to methionine salvage in *R.* *rubrum* and *B.* *subtili*s. Our approach revealed elaborate metabolic strategies used by microbes to cope with stressful environments (e.g., MTA feeding) through the coordinated regulation of several expected and unexpected metabolic pathways.

## Materials and methods

### Bacterial strains and growth conditions

*Bacillus subtilis* 168 (a gift from the laboratory of G. Ordal) was grown aerobically in either Luria-Bertani broth (Albers 2009) or minimal medium at 37 °C as previously described (Sekowska and Danchin 2002). *R.* *rubrum* (DSM 467, ATCC 11170, American Type Culture Collection, Manassas, VA, USA) and its MTXu 5-P methylsulfurylase mutant (a gift from R. Tabita and Jaya Singh) were grown aerobically in the dark at 30 °C on 20–2,000 mL minimal medium with sulfate or MTA as the sole sulfur sources, as previously described (Erb et al. 2012). See the Supplementary methods for details.

### LC–Fourier transform (FT) MS metabolomics

Cell suspensions (OD_600_ = 6 for *B.* *subtilis*, OD_578_ = 6 for *R.* *rubrum*) were incubated at optimum growth temperature (temperature = 30 °C) in minimal media without a sulfur source (control samples) or with 1 mM MTA (feeding experiments). Sampling was carried out at various time points (*B.* *subtilis*: 0, 2, 5 and 15 min; *R.* *rubrum*: 0, 10 and 20 min) according to the strains. Then cells were pelleted and immediately frozen in liquid nitrogen. Metabolites were extracted from the frozen cell pellets by resuspending with 0.375 mL 10 mM ammonium bicarbonate buffer (pH 9.2) containing 90 % acetonitrile. LC–FTMS analysis was carried out using an 11T LTQ-FT Ultra mass spectrometer (Thermo-Fisher Scientific, Waltham, MA, USA) equipped with an Agilent 1200 HPLC system in negative mode (Agilent Technologies, Santa Clara, CA, USA) (Evans et al. 2011). See the Supplementary methods for details.

### LC–FTMS data analysis

Data analysis was carried out with the analytical platform as described in the Supplementary methods. This discovery platform consists of various functional modules to cope with rising issues in untargeted metabolomics, including: data pre-processing, isotope pattern analysis, molecular formula determination, database searching and pathway activity profiling. Peaks were detected using XCMS (http://metlin.scripps.edu/xcms/). Peak lists were filtered to remove adducts and isotopic peaks. Molecular formulas were determined by comparing experimental isotopic patterns with theoretically predicted isotopic patterns modeled with Bayesian statistics. Seed metabolites were automatically detected based on raw LC–MS data. Both monoisotopic masses and the top three predicted molecular formulas were searched against the Kyoto Encyclopedia of Gene and Genomes (KEGG) (Kanehisa et al. 2012) database to putatively annotate peaks and to build-up metabolite sets using pathway information. Constructed metabolite sets (i.e., implicated pathways) were evaluated using the MSEA approach with a Kolmogorov–Smirnov running sum statistic (Mootha et al. 2003). Highly perturbed but not annotated peaks were listed for further identification experiments. See Supplementary methods for details.

### Quantitative real time polymerase chain reaction (qRT-PCR)

qRT-PCR was performed as previously described (Pfaffl 2001). Cells were harvested at OD = 0.6, and RNA was extracted using an RNeasy protect kit (Qiagen, Gaithersburg, MD, USA) according to the manufacturer’s recommendations. Total RNA was resuspended in PCR-grade nuclease-free water, and RNA quality and concentration were estimated by optical density measurement using a Nanodrop 2000 spectrophotometer (Fisher Scientific, Pittsburgh, PA, USA). Each sample of 500 ng total RNA was reverse transcribed using a First Strand cDNA Synthesis Kit (Fermentas, Pittsburgh, PA, USA). Real-time PCR reactions were carried out on a LightCycler 480 (Roche, Indianapolis, IN, USA) using the SYBR Green detection format. Changes in the expression were calculated relative to the expression of 16SrRNA. After each PCR run, a melting curve analysis was carried out to control for production of primer dimers and/or non-specific PCR products. Expression levels of mRNA were estimated using external standard curves with serially diluted plasmids/PCR products with known concentrations for each target gene. Fold changes in mRNA expression during treatment were calculated using the crossing point (Cp) for each sample and the efficiency (Eff) of each transcript using the formula (Eff_target_ gene)^ΔCp^/(Eff_housekeeping_ gene)^ΔCp^. The fold changes were estimated relative to 16SrRNA.

### RNAseq and analysis

Subtraction of ribosomal RNAs from a 10 µg total RNA sample using an Ambion MICROBExpress Kit (Applied Biosystems, Foster City, CA, USA), as well as subsequent sequencing of the enriched mRNA fraction (Illumina, San Diego, CA, USA, 100 bp single end, directional RNAseq method) were performed at the W.M. Keck Center for Comparative and Functional Genomics (University of Illinois at Urbana-Champaign) using in-house protocols. The reads were on average between 70 and 80 nt, at a total of about 22 million reads for the RNA preparation from sulfate-grown cells and about 20 million reads for the RNA preparation from MTA-grown cells. RNAseq data were aligned against the *R.* *rubrum* genome (Accession number NC_007643) and analyzed using the CLC genomics workbench software, version 3.7 (CLC bio, Cambridge, MA, USA) according to the user’s manual. Briefly, short reads were aligned against the fully sequenced genome of *R.* *rubrum* to determine unique and total gene reads.

## Results

### Analytical strategy

**Fig. 1:**
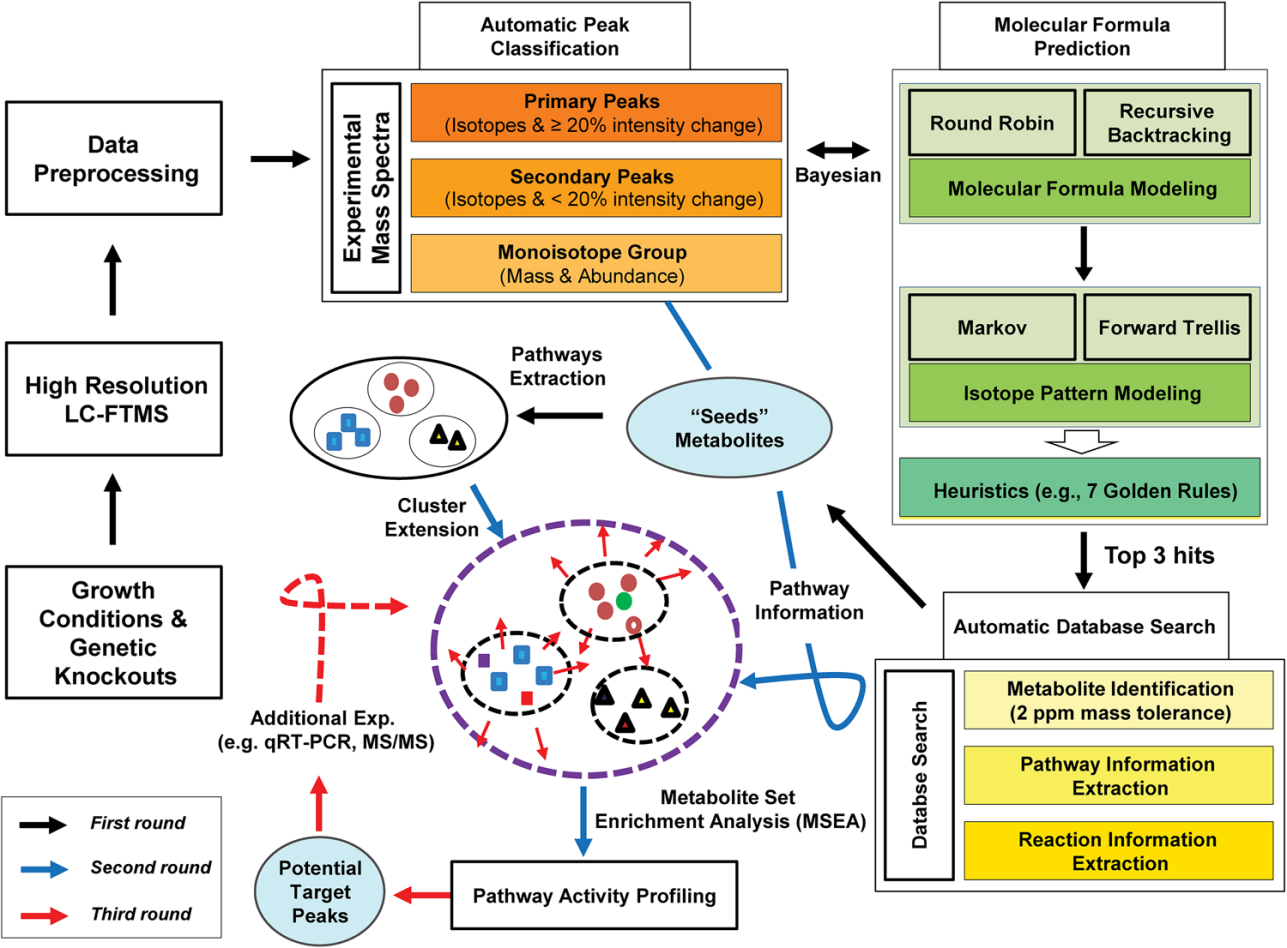
An overview of our analytical strategy. Specific changes in bacterial growth conditions and genetic knockout yield high resolution LC–MS data from which active pathways are detected by computational analysis and experimental validation, employing metabolite profiling, nominating altered metabolites, modeling molecular formulas, evaluating pathway activities and validating detected active pathways with qRT-PCR and RNAseq. *Black*, *blue* and *red* *arrows* indicate chronology of events in the workflow

Although there has been great progress in LC–MS-based metabolomics that enables one to extract biological insight from metabolite profiles, more remains to be done. Building upon existing technologies, we developed a bioinformatics platform that includes various functional modules: data pre-processing (including chromatographic feature detection, deconvolution and filtering), automatic mass spectral peak grouping (peaks with isotopic patterns versus peaks without isotopic patterns), molecular formula determination, database searching and pathway activity evaluation based on MSEA (see Supplementary Material, Source Codes.zip). This novel approach enabled a streamlined process for the detection of actively changing metabolic pathways from raw LC–MS data (see Supplementary Material Fig. S1, for system design). Mass spectral features refined by the data pre-processing module were classified into three groups. The primary group contained features with visible isotope peaks and with a >20 % abundance change between the experiment and control. The secondary group contained features with visible isotope peaks, but whose abundance change between groups was <20 %. The tertiary peak group had features with no visible isotopic pattern. Molecular formulas were determined for peaks in the primary and secondary groups using well-known round-robin and recursive backtracking algorithms (Bocker et al. 2008, 2009; Bocker and Liptak 2007). During this process, non-biological molecular formulas were further filtered using previously published heuristics (i.e., the seven golden rules) (Kind and Fiehn 2007). Theoretical isotopic patterns for predicted molecular formulas were modeled by the first-order Markov process and the forward trellis algorithm (Snider 2007). Next, these simulated isotopic patterns were compared to experimental isotopic patterns based on Bayesian statistics. Only the top three candidates identified by this process were searched against the KEGG metabolite database with 5 ppm mass tolerance. Hits in KEGG were considered as “seed metabolites”, a major concept of this strategy. Thus, seed metabolites are defined as metabolites that show significant abundance changes (>20 %), clear isotopic patterns, and returned search hits in a database; these provided the basis (“seeds”) for the annotation of mass spectral peaks that were not otherwise annotated with high confidence. Among search hits using mass alone against the KEGG database, only hits in the same pathways as those of the seed metabolites were considered as putative candidates for annotation. These candidates were clustered into their implicated pathways and then used as MSEA. Peaks that were highly perturbed (>20 % abundance changes) without search hits in public databases (due to coverage issues) (Tautenhahn et al. 2012) were queued for more elaborate annotation experiments. The details of the analytical flow are depicted in Fig. 1. Detected active pathways were further validated by transcriptomics.

### Data pre-processing

A basic and mandatory step in metabolite profiling is to reduce data complexity caused by adducts, isotopologues, multimers and signals arising from chemical and electronic noise. Currently, this feature refinement is carried out through either a manual or semi-automated process that mostly deals with the features of biological interest and not the complete set of detected features (Dunn 2008). While there are several publicly available LC–MS based bioinformatics tools for peak detection and alignment, including XCMS (Smith et al. 2006) and MZmine (Katajamaa and Oresic 2005), there are few public tools for this error-prone and time-consuming raw data refinement process (Alonso et al. 2011).

**Fig. 2:**
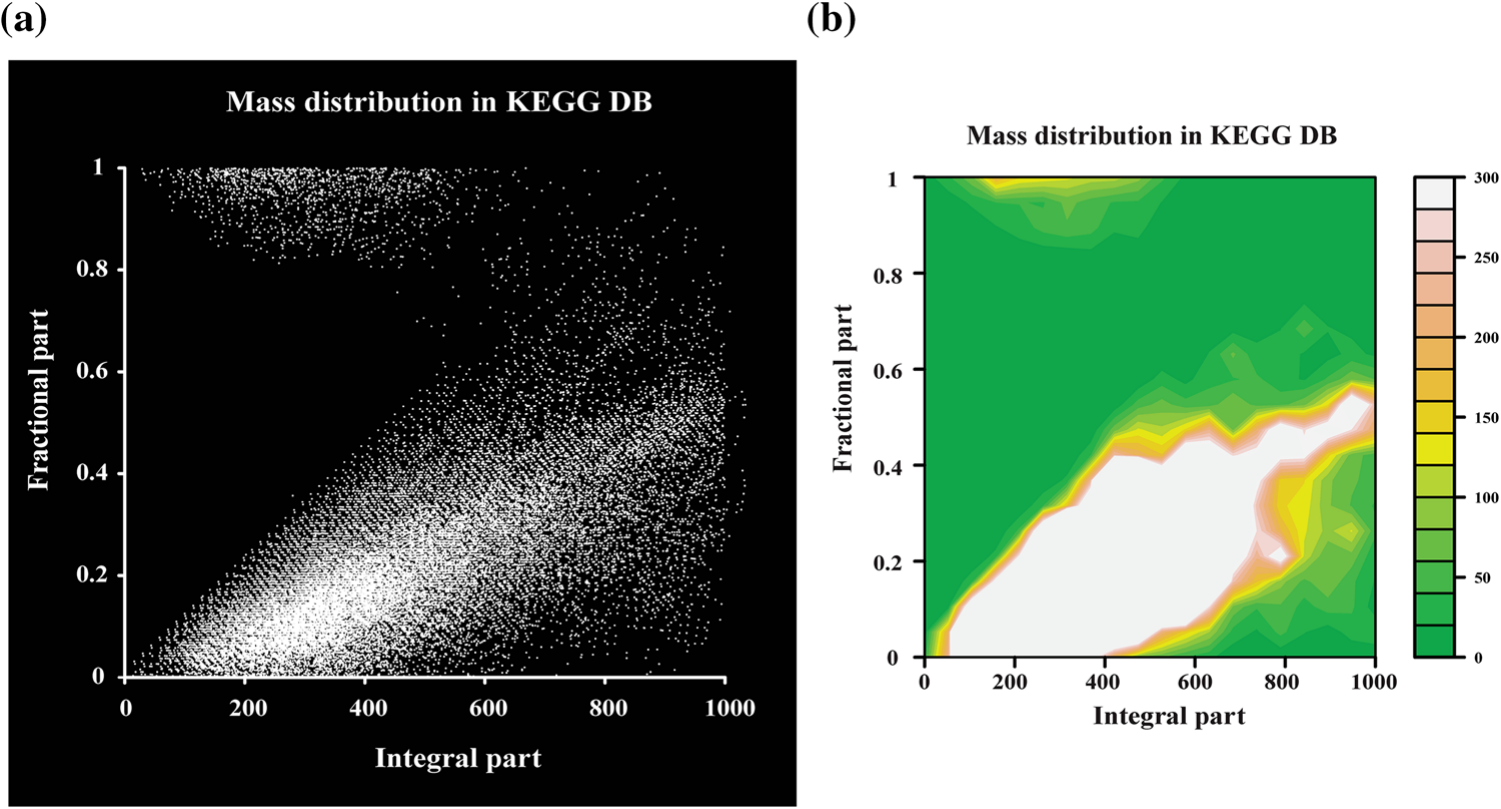
Mass distribution (integral part vs. fractional part) to eliminate non-biological signals in the KEGG database. **a** Scatter plot of mass distribution in KEGG. **b** Contour map of mass distribution in KEGG. The mass distribution between the integral parts and fractional parts of the monoisotopic masses was contrasted against KEGG. Inorganic metal salts and polyhalogenated molecules were eliminated manually. The distribution clearly shows that biological molecules mainly occupy two specific regions in the distribution space, as the contour map shows that only a small portion of molecules (<0.3 %) appeared outside of these regions

In this study, a mass difference matrix was constructed through an all-by-all mass comparison among detected peaks to filter the redundant data and to recognize isotopic patterns. For each peak, possible adducts and multimers were eliminated by comparing their masses with the entire mass differences in the matrix. Isotopic patterns (^13^C, ^15^N, ^18^O, ^34^S) were also analyzed based on mass difference and retention time (RT) with tolerances (e.g., mass tolerance = 2 ppm and RT tolerance = 60 s) and stored as a list of matrices for molecular formula determination. Then, isotopologues (except monoisotopes) were removed from the peak list. We took notice of the limited usage of elements in the biological molecular formula in a previous study, conceptualized as the seven golden rules (Kind and Fiehn 2007). This narrow elemental composition constraint has an effect that is observable in the accurate mass, the mass defect, which is defined as the difference between the nominal and exact (Zhang et al. 2009). This prior knowledge inspired us to investigate whether there is a biased distribution between the integral part and the fractional part of the masses of biological molecules. We examined the mass distribution of all masses in the KEGG database (May 16, 2011 version) and noticed that there was a clear region that was not occupied by metabolites due to the limited elemental compositions, as shown in Fig. 2. This trend was also observable in the PubChem compound database (June 12, 2012 version), in which many synthetic compounds were included (Supplementary Fig. S2). Even in PubChem, only <0.3 % of data were in this limited region, and most data were in the high-density region. Consequently, this mass distribution was used as a non-biological signal filter in our analysis at the pre-processing step, eliminating up to 18 % of the initially detected features (Table 1). After deconvoluting the peaks (e.g., isotopologues, adducts, multimers and non-biological signals), we were able to greatly reduce the number of features from the raw LC–MS data. Greater than 50 % of the initially detected features in both organisms were excluded from further analysis. The distribution of features according to the data pre-processing is shown in Table 1. Notably, features with determined elemental compositions occupy only a small portion of the detected features (9–12 % of *R.* *rubrum* and 5–6 % of *B.* *subtilis*), suggesting further technical improvements are needed to detect isotopologues in untargeted metabolomics.

**Table 1:** Distribution of putatively annotated peaks according to the time points (negative mode, 100–1,000 *m/z*)

Feature summary	*R.* *rubrum* (min)		*B.* *subtilis* (min)		
	10	20	2	5	15
No. of features	2,294	4,205	4,963	4,416	4,322
Invalid RT (e.g. 120 s ≤ RT ≤ 2,030 s)	646	1,565	1,035	989	1,047
No. of artifact features (e.g., adducts and multimers)	82	126	207	168	154
No. of non-biological signals (i.e., mass distribution filter)	145 (6.3 %)^a^	347 (8.3 %)^a^	704 (14.2 %)^a^	672 (15.2 %)^a^	620 (14.3 %)^a^
No. of isotopes	339	434	455	387	368
Total no. of eliminated features	1,212	2,472	2,401	2,216	2,189
No. of refined features for further analysis	1,082 (47.1 %)^a^	1,733 (41.2 %)^a^	2,562 (48.4 %)^a^	2,200 (49.8 %)^a^	2,133 (49.4 %)^a^
No. of predicted molecular formulas	270 (11.8 %)^a^	366 (8.7 %)^a^	252 (5.1 %)^a^	240 (5.4 %)^a^	225 (5.2 %)^a^

^a^The figures are expressed as a percentage of all initially detected features

### Seed metabolites and dynamic build-up of metabolite sets

A challenge in metabolite profiling is the effort to automate the annotation of the detected peaks with high confidence, high coverage and low false positives in order to identify active pathways and extract biological insights. A simple search against publicly available databases with only nominal mass information produces many false positives. As an alternative, accurate mass and elemental composition constraints are used together to define metabolites by determining their molecular formulas (Bocker et al. 2008, 2009; Rogers et al. 2009; Brown et al. 2011). However, only a small portion of the LC–MS data contains this useful information, so the problem of limited coverage remains to be solved. Furthermore, since metabolite profiling is a snapshot of metabolism at a specific time point, not all of the possible metabolites are detected, and slightly different subsets of metabolites of a metabolic pathway can be observed even under the same experimental conditions. To apply enrichment analysis to detect unexpectedly interwoven pathways, biologically meaningful metabolite sets should be constructed exhaustively.

**Fig. 3:**
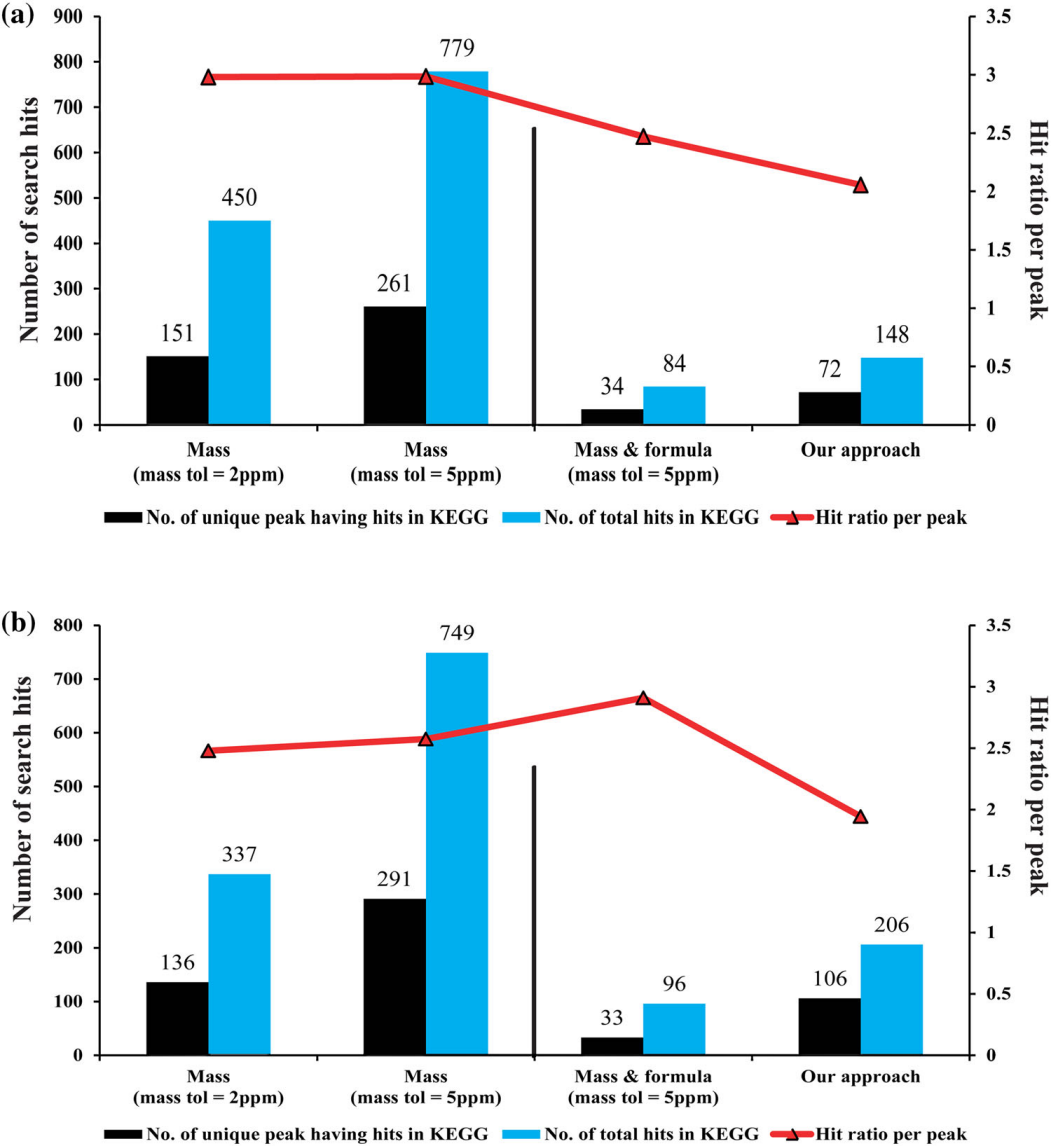
Performance evaluation of putative peak annotation of **a** *R.* *rubrum* at 20 min, **b** *B.* *subtilis* at 2 min. The performance of the putative peak annotation was indirectly evaluated by introducing a search HRPP, calculated by dividing the total number of hits in the KEGG database by the total number of input peaks. The *black bars* represent the total number of unique input peaks, and the *blue bars* represent the total number of search hits. The *red line* represents the performance as evaluated by HRPP. Two mass tolerances (2 and 5 ppm) were used for the simple search (*bars on the left* *of* the *black line*), and for others, a 5 ppm mass tolerance was used (*bars on the left of the black line*)

To cope with these challenges, we used seed metabolites and the dynamic build-up of metabolite sets (see Fig. 1). Also, we integrated several analytical processes to reduce the total number of metabolite sets to be evaluated. The peaks in the primary group were searched against the KEGG database using molecular formulas and monoisotopic masses. Search hits provided our initial seed metabolites and their pathway information was extracted. The extracted pathways were used to assign peaks for the secondary group and the tertiary group by searching against the KEGG database. For the secondary group, molecular formulas were used together with pathway information. Search hits from this round were also added to the existing pathway clusters. In this way, metabolite sets for the implicated pathways were dynamically built-up and evaluated by the enrichment analysis based on the MSEA procedure. To calculate the performance (e.g., confidence and coverage) of these putative annotations before validation, a simple measure called the hit ratio per peak (HRPP) was introduced to compare the performance indirectly. The HRPP is the ratio of the number of total hits in the database divided by the number of peaks having at least one hit in the database. Performance is inversely related to the HRPP. As shown in Fig. 3, simple searches based only on accurate mass produced relatively large numbers of search hits, but also increased the HRPP (e.g., HRPP = 2.98 at 20 min, *R.* *rubrum*; and HRPP = 2.57 at 2 min, *B.* *subtilis*, using a 5 ppm mass tolerance). Isotopic abundance patterns are known to be useful in reducing the number of potential elemental compositions and providing high confidence to the search hits (Kind and Fiehn 2006). Although elemental composition was used for filtering the database searches, and search hits were further reduced by eliminating false positives, the HRPPs fluctuated in our experiment (e.g., HRPP = 2.47 at 20 min, *R.* *rubrum*; and HRPP = 2.82 at 2 min, *B.* *subtilis*, using a 5 ppm mass tolerance). This was somewhat unexpected, because higher confidence is expected at the expense of coverage when combining mass and molecular formula information. However, the unavoidable incompleteness of available databases, and variable quality of the LC–MS data, may have affected the HRPP. In contrast, our approach consistently showed higher confidence and coverage, supporting the usefulness of the seed metabolite approach (e.g., HRPP = 2.05 at 20 min, *R.* *rubrum*; and HRPP = 1.89 at 2 min, *B.* *subtilis*, using a 5 ppm mass tolerance). Details are shown in Fig. 3, and Supplementary Material Tables S1 and S2.

### Actively changing metabolic pathways detected by the enrichment analysis

Dynamically constructed metabolite sets (i.e., implicated pathways) were further curated into subcategories manually and evaluated by the enrichment analysis process to detect interconnected pathways upon metabolic perturbation with MTA, a key metabolite in bacterial methionine salvage pathways.

**Fig. 4:**
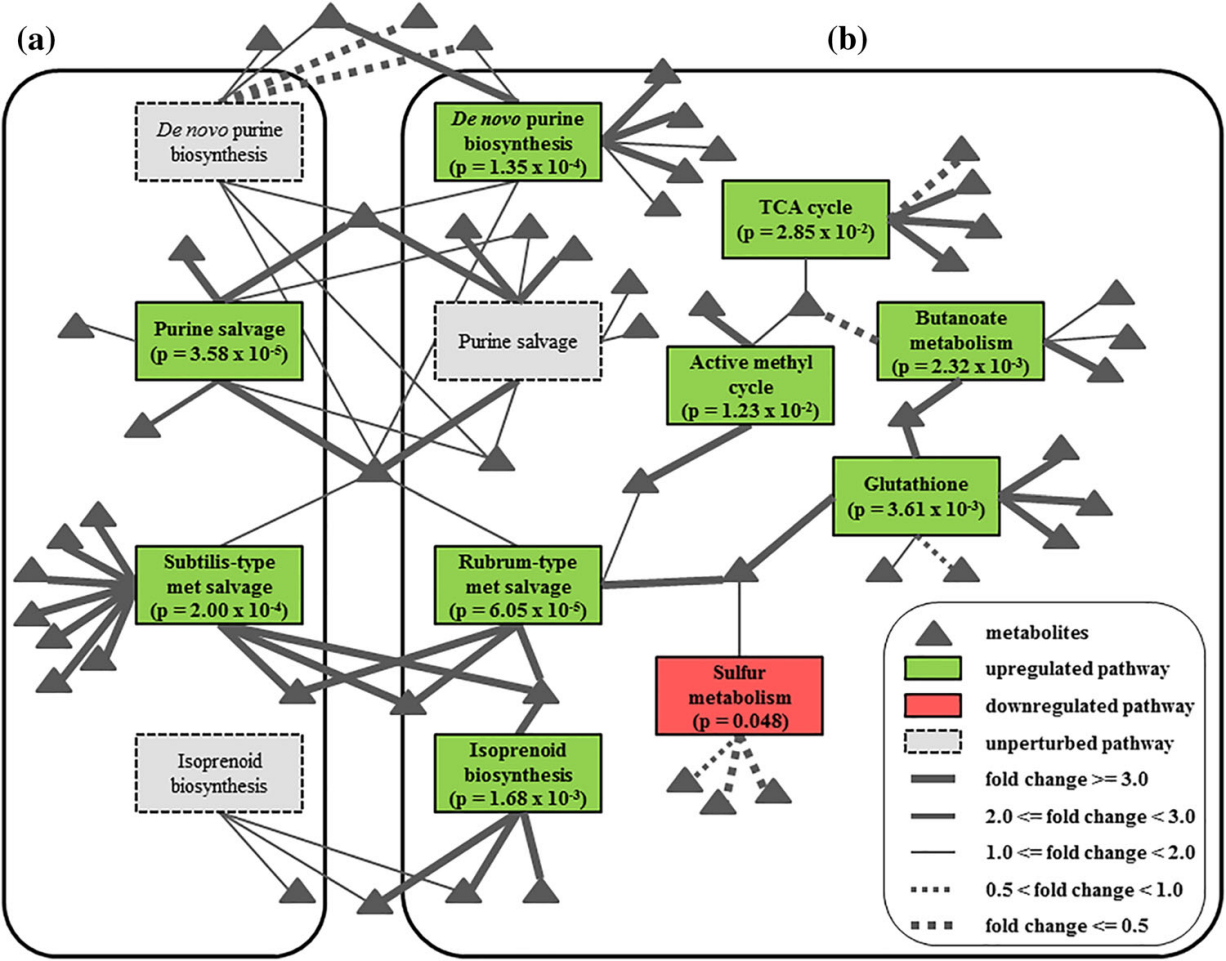
Actively changing metabolic pathways detected from implicated pathways via untargeted metabolomics. Actively changing metabolic pathways in **a** *B.* *subtilis* and **b** *R.* *rubrum* detected by untargeted metabolomics. In *B.* *subtilis*, the subtilis-type methionine salvage pathway and the purine salvage pathway were detected as active pathways upon MTA feeding. In *R.* *rubrum*, eight total metabolic pathways were detected as active pathways upon MTA feeding, including the rubrum-type methionine salvage pathway, the AMC, the sulfur metabolism, the isoprenoid pathway, the purine metabolism, the TCA cycle, the glutathione metabolism and the butanoate metabolism. *Rectangles* implicated pathways; *triangles* metabolites detected by LC-FTMS. *Green* up-regulation of the corresponding metabolites in the pathways. *Red* down-regulation of the metabolites. *Gray* pathways that are not much affected upon MTA perturbation. *Line style* indicates the fold change of the metabolites

In *B.* *subtilis*, a total of 26 implicated pathways were generated based on the concept of seed metabolites and the dynamic build-up process. Among them, only the purine salvage pathway (*p*-value = 3.58 × 10^−5^ at 2 min) and the classical, subtilis-type methionine salvage pathway (*p*-value = 2.00 × 10^−4^ at 2 min) were characterized as active pathways using the enrichment analysis approach (Fig. 4a). Fold changes and corresponding *p*-values are detailed in Supplementary Table 3. Adenine and hypoxanthine were clearly up-regulated upon MTA perturbation. The activation of the purine salvage pathway is in line with the cleavage of adenine from MTA. However, metabolites in the de novo purine biosynthesis pathway were not significantly affected. Xanthine and xanthosine-5′-phosphate were down-regulated, but the levels of adenosine-5′-monophosphate and 5′-phosphoribosyl-5-amino-4-imidazolecarboxamide were not changed. Similar to the purine salvage pathway, the subtilis-type methionine salvage pathway was also determined to be active, even though some metabolites were not accumulated according to the time points (e.g., 2,3-diketo-5-methyl-thiopentenyl-1-phosphate, 2-hydroxy-3-keto-5-methylthiopentenyl-1-phosphate and 4-methylthio-2-oxobutanoate) or detected accordingly in our experimental setup (e.g., 1,2-dihydroxy-3-keto-5-methyl-thiopentene and 3-methylthiopropionate).

In *R.* *rubrum*, a total of 53 implicated pathways were dynamically constructed. Among them, eight were considered as active pathways using the enrichment analysis approach (Fig. 4b). As expected, the purine salvage pathway (*p*-value = 1.35 × 10^−4^ at 20 min), the rubrum-type methionine salvage pathway (*p*-value = 6.05 × 10^−5^ at 20 min), and the non-mevalonate isoprenoid pathway (*p*-value = 1.68 × 10^−3^ at 20 min) were clearly affected by MTA feeding. Thus, the unexpected link of MTA metabolism with the isoprenoid pathway in *R.* *rubrum*, as recently published by our group (Erb et al. 2012), was re-confirmed by this analysis, establishing the MTA-isoprenoid shunt as an essential part of the novel MTA recycling strategy in *R.* *rubrum*. Note that isoprenoid biosynthesis is not affected in *B.* *subtilis* upon MTA feeding, which is in line with the classical methionine pathway in this organism. The fold changes and *p*-values of detected metabolites in the isoprenoid biosynthesis of *R.* *rubrum* were compared with those of *B.* *subtilis*, with results shown in Supplementary Material Table S4.

**Fig. 5:**
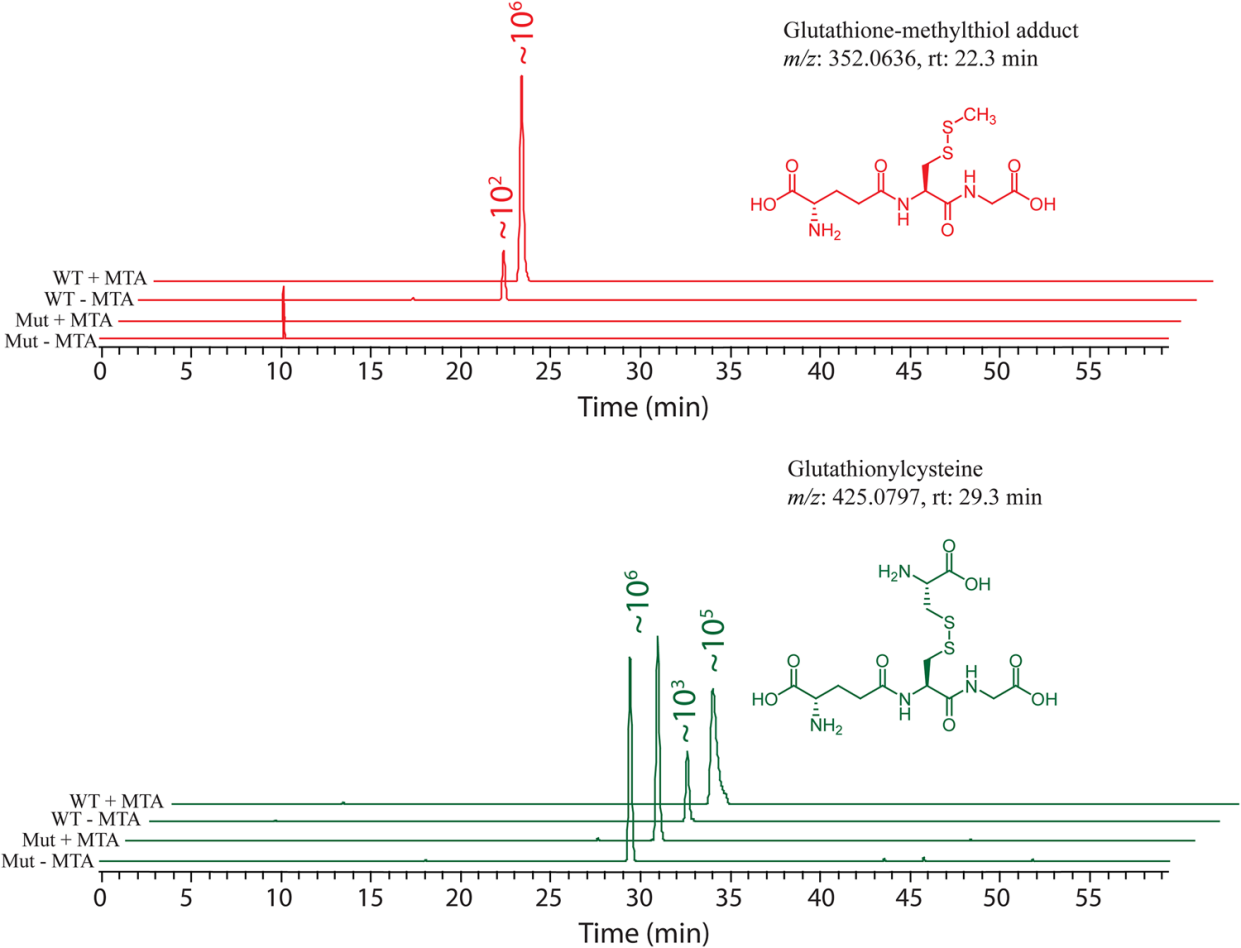
Abundance changes of glutathione-related compounds in metabolomics. Glutathione-related peaks in metabolomics at 10 min (*R.* *rubrum*) are shown. Notably, the glutathione–methylthiol adduct (*m/z*: 352.0636, RT: 22.3 min) is highly induced by MTA feeding in the wild-type organism but is not induced in the MTXu 5-P methylsulfurylase mutant. This adduct provides strong in vivo evidence that glutathione functions as a coenzyme in MTXu 5-P methylsulfurylase-catalyzed dethiomethylation, a novel route to connect rubrum-type methionine salvage to the isoprenoid pathway. *WT* wild type, *Mut* MTXu-5P methylsulfurylase mutant

In *R.* *rubrum*, the rubrum-type methionine salvage pathway was also strongly intertwined with the active methyl cycle (AMC) (*p*-value = 1.23 × 10^−2^), as evidenced by the strong up-regulation of *S*-adenosyl-l-homocysteine (SAH) (fold change = +702, *p*-value = 4.72 × 10^−14^) and SAM (fold change = +148, *p*-value = 6.22 × 10^−7^) at 10 min. However, metabolites of the AMC were down-regulated at 20 min. Similar to the AMC, glutathione metabolism was also up-regulated (*p*-value = 3.61 × 10^−3^) at 10 min and down-regulated at 20 min at the level of metabolites. Notably, we observed that a glutathione-methylthiol adduct (*m/z* = 352.0636, RT = 22.3 min) was highly and consistently up-regulated by adding MTA to wild-type *R.* *rubrum* (fold change = +914, *p*-value = 1.35 × 10^−11^ at 10 min; fold change = +1,573, *p*-value = 1.05 × 10^−4^ at 20 min). However, this compound was not observed in the MTXu 5-P methylsulfurylase mutant (see Fig. 5). Additional in vitro and in vivo feeding experiments confirmed the identity of glutathione–methylthiol adduct (Supplementary Figs. S3, S4). This glutathione–methylthiol adduct is strong evidence that glutathione functions as a coenzyme in vivo in MTXu 5-P methylsulfurylase-catalyzed dethiomethylation. Glutathione itself was found to be unstable during our sample preparation and analysis; therefore, we do not report changes in this metabolite here. Finally, the tricarboxylic acid (TCA) cycle showed a similar pattern to the AMC and glutathione metabolism upon MTA feeding (*p*-value = 2.85 × 10^−2^), with an up-regulation at 10 min and a down-regulation at 20 min. Malate (fold change = +11; *p*-value = 2.20 × 10^−10^), 2-oxoglutarate (fold change = +270; *p*-value = 1.91 × 10^−12^) and fumarate (fold change = +8; *p*-value = 4.59 × 10^−12^) were highly up-regulated with statistical significances at 10 min. Butanoate metabolism, which involves these metabolites and downstream metabolites of the TCA cycle, was also up-regulated at 10 min (*p*-value = 2.32 × 10^−3^), and sulfur metabolism was down-regulated at 20 min (*p*-value = 0.048). The fold changes and *p*-values of their constituent metabolites in these active pathways are listed in Supplementary Material Table S5.

### Validation by qRT-PCR

Subsets of genes detected in each active pathway have been manually validated upon MTA perturbation. Selected genes and primers are listed in Supplementary Material Table S6. After feeding MTA, gene expression levels were monitored at selected time points by qRT-PCR. In *B.* *subtilis*, genes of the classical, subtilis-type methionine and purine salvage pathways were investigated based on their activation observed upon MTA perturbation (Supplementary Material Table S7). In line with the metabolomics results, genes in the subtilis-type methionine salvage pathway were highly up-regulated by MTA perturbation (BSU27270 = +5.9, BSU13560 = +4.4, BSU13550 = +9.5 and BSU13620 = +5.4). However, genes in the purine salvage pathway were not induced by MTA feeding, even though RNAs were detected under the growth conditions, suggesting that accumulation of metabolites in these pathways in vivo is not related to up-regulation of transcription.

As listed in Supplementary Material Table S8, in *R.* *rubrum*, genes in the rubrum-type methionine salvage pathway (e.g., Rru_A0361, Rru_A0360, Rru_A1998, Rru_A2000, Rru_A0774 and Rru_A0784) and in the isoprenoid pathway (e.g., Rru_A1592 and Rru_A0263), were highly up-regulated upon MTA feeding. Up-regulation of the MTXu 5-P methylsulfurylase gene (Rru_A2000, fold change = +3.1 at 20 min) confirms its critical role in the novel MTA isoprenoid shunt. Similarly, the gene encoding the methylthioribulose-1-phosphate isomerase, a RuBisCO-like protein that provides the substrate for the MTXu 5-P methylsulfurylase, was also highly affected (Rru_A1998, fold change = +4.3 at 20 min) upon MTA perturbation. Notably, 1-deoxy-d-xylulose-5-phosphate synthase (*dxs*, Rru_A2619, fold change = −8.8 at 20 min), which converts d-glyceraldehyde-3-phosphate into DXP, was suppressed by MTA-feeding. Since this gene was expressed prior to MTA perturbation, the qRT-PCR results suggest that the non-mevalonate pathway is the major route to isoprenoid biosynthesis under physiological conditions. However, upon MTA perturbation, cells suppressed the use of the non-mevalonate pathway and instead activated the rubrum-type methionine salvage pathway to channel the carbon skeleton of methylthioxylulose-5-phosphate into DXP, indicating that the MTA-isoprenoid shunt is able to contribute significantly to DXP synthesis in *R.* *rubrum*. Expression levels of genes were stabilized to approximately basal levels after 60 min. Although the isoprenoid pathway intermediates were not affected in *B.* *subtilis* upon MTA perturbation, two genes of the non-mevalonate isoprenoid pathway were analyzed in their expression pattern to rule out the possibility that isoprenoid genes are induced in *B.* *subtilis*. Expression of the two marker genes was not induced, in line with the idea that methionine salvage and isoprenoid biosynthesis are indeed unlinked in *B.* *subtilis* (BSU24270 = +1.4, BSU16550 = −2.9) (see Supplementary Material Table S7).

In contrast to the genes involved in methionine salvage and isoprenoid biosynthesis in *R.* *rubrum*, genes of the purine salvage pathway (Rru_A2483, Rru_A0149 and Rru_A0607) and de novo purine biosynthesis (Rru_A2168, Rru_A1963, Rru_A0299 and Rru_A3655) were expressed constitutively. As observed for *B.* *subtilis*, these genes are apparently expressed under physiological conditions and are not affected by MTA perturbation.

Our qRT-PCR data show that the active pathways detected using our metabolomics analysis platform are reliable, whether they are accompanied by a change of gene expression upon MTA feeding, or by consistent expression of genes under growth conditions before MTA is added (e.g., de novo purine biosynthesis). qRT-PCR allows sensitive and specific assays for targeted genes, but it covers only a limited number of genes in the whole genome. We also felt it was important to clarify both the role of the *trans*-sulfuration pathway and the sources and sinks of glutathione in the context of *R.* *rubrum* MTA feeding; this required that the expression patterns of many more genes be checked. Hence, an RNAseq experiment was conducted for *R.* *rubrum*.

### Assembling missing pieces of the puzzle by RNAseq

The RNAseq (Supplementary Material Table S9) clearly reconfirmed our qRT-PCR results for *R.* *rubrum*, even though there were sensitivity differences between the two techniques. In addition, RNAseq provided some clues for the role of *trans*-sulfuration and for the multiple fates of methanethiol liberated during rubrum-type MTA salvage. RNAseq showed that glutathione metabolism was perturbed by MTA feeding, as evidenced by NADPH-glutathione reductase (Rru_A0682, fold change = +2.4) and glutathione *S*-transferase (Rru_A0332, fold change = +3.7). In line with the metabolomics data from this study (i.e., glutathione–methylthiol adduct), these gene expression patterns describe well the biochemistry of glutathione. Cysteine can also be metabolized to hydrogen sulfide and pyruvate by cystathionine β-synthase (CBS) and cystathionine γ-lyase (CGL) (Singh et al. 2009). Although metabolites in pyruvate metabolism were not detected via our LC–MS based metabolomics, there were several up-regulated genes in this pathway, including hydroxyacylglutathione hydrolase (Rru_A2371, fold change = +2.74), acetate kinase (Rru_A2998, fold change = +2.51), aldehyde dehydrogenase (Rru_A0931, fold change = +1.69), acetyl-CoA acetyltransferase (Rru_A0274, fold change = + 1.65) and formate acetyltransferase (Rru_A3000, fold change = +1.84).

## Discussion

We describe an analytical strategy that combines untargeted metabolomics and transcriptomics (i.e., a combination of targeted qRT-PCR and RNAseq) to decipher intertwined metabolic pathways of universal application. The LC–MS platform we developed facilitates the data analysis and includes several critical functional modules: data processing, isotope pattern analysis, molecular formula determination, database searching and pathway activity profiling. Together with the concepts of seed metabolites and the dynamic build-up of metabolite sets, this platform enables a streamlined process for detecting interconnected metabolic pathways from raw LC–MS data. Implicated pathways and their constituent metabolites are automatically annotated with high confidence and coverage by integrating different sources of information. Statistical evaluation using MSEA confers high confidence to the annotations of perturbed metabolites and their respective pathways.

**Fig. 6:**
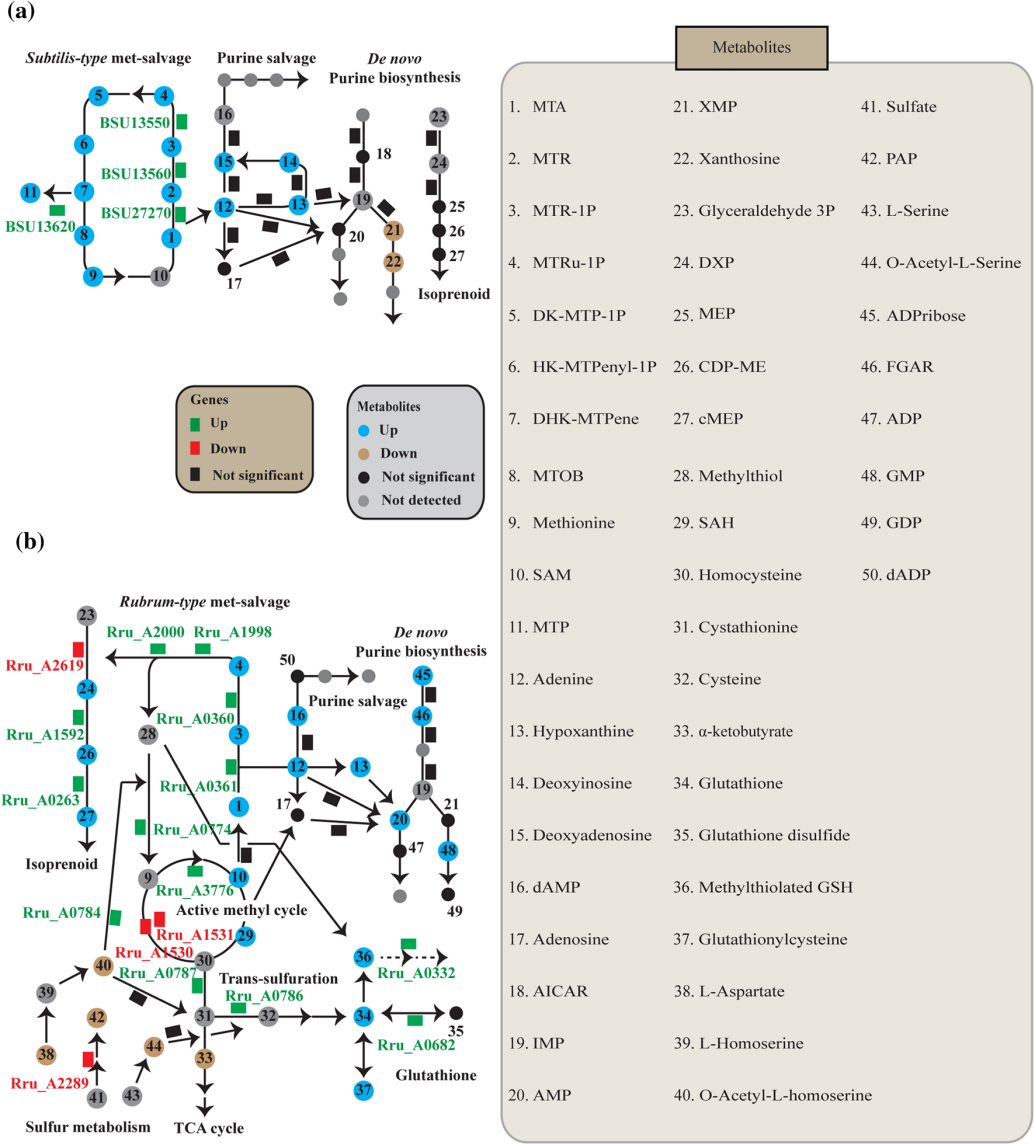
A coordinated response of metabolic pathways upon MTA perturbation. Actively changing metabolic pathways upon MTA perturbation in **a** *B.* *subtilis* and **b** *R.* *rubrum.* Genes are represented by the prefixes, *BSU* (*B.* *subtilis*) and *Rru* (*R.* *rubrum*). *Rectangles* genes; *Circles* metabolites. The *numbers in the circles* correspond to the following metabolites: *1* MTA, *2* 5-Methylthio-d-ribose, *3* *S*-Methyl-5-thio-d-ribose 1-phosphate, *4* *S*-Methyl-5-thio-d-ribulose 1-phosphate, *5* 2,3-Diketo-5-methyl-thiopentyl-1-phosphate, *6* 2-Hydroxy-3-keto-5-methylthiopentenyl-1-phosphate, *7* 1,2-Dihydroxy-3-keto-5-methylthiopentene, *8* 4-Methylthio-2-oxobutanoate, *9* Methionine, *10* SAM, *11* 3-Methylthiopropionate, *12* Adenine, *13* Hypoxanthine, *14* Deoxyinosine, *15* Deoxyadenosine, *16* Deoxyadenosine monophosphate, *17* Adenosine, *18* 5-Amino-1-(5-phospho-d-ribosyl) imidazole-4-carboxamide, *19* Inosine monophosphate, *20* Adenosine 5′-monophosphate, *21* Xanthosine 5′-phosphate, *22* Xanthosine, *23* Glyceraldehyde 3-phosphate, *24* DXP, *25* 2-C-Methyl-d-erythritol 4-phosphate, *26* 2-Phospho-4-(cytidine 5-diphopho)-2-C-methyl-d-erythritol, *27* 2-C-Methyl-d-erythritol 2,4-cyclodiphosphate, *28* Methanethiol, *29* *S*-Adenosyl-l-homocysteine, *30* Homocysteine, *31* Cystathionine, *32* Cysteine, *33* α-Ketobutyrate, *34* Glutathione, *35* Glutathione disulfide, *36* Methylthiolated glutathione, *37* Glutathionylcysteine, *38* l-Aspartate, *39* l-Homoserine, *40* *O*-Acetyl-l-homoserine, *41* Sulfate, *42* Phosphoadenosine phosphate, *43* l-Serine, *44* *O*-Acetyl-l-serine, *45* Adenosine diphosphate ribose, *46* 5′-Phosphoribosyl-*N*-formylglycinamide, *47* Adenosine 5′-diphosphate, *48* Guanosine monophosphate, *49* Guanosine diphosphate, *50* 2′-Deoxyadenosine 5′-diphosphate). Three colors are used to represent the genes. *Green* up-regulation; *Red* down-regulation and *Black* genes showing no big changes in their expression levels. Four colors are used to represent the metabolites. *Blue* up-regulation; *Brown* down-regulation; *Black* no change; *Gray* metabolites that were not detected using LC–FTMS. As compared to *B.* *subtilis*, *R.* *rubrum* has an elaborate metabolic strategy to cope with a stressful environment (e.g., utilization of MTA as the sole sulfur source) through the coordinated regulation of metabolic pathways. See text for detailed discussion

We applied this analytical strategy to unravel metabolic pathways linked to methionine salvage in *R.* *rubrum* and *B.* *subtili*s by combining changes in bacterial growth conditions (e.g., MTA as a sole sulfur source) and a genetic knockout. Our results revealed the coordinated regulation of several expected and unexpected metabolic pathways when MTA was provided to either organism (Fig. 6). As expected, the classical, subtilis-type methionine salvage pathway and the purine salvage pathway were active in *B.* *subtilis* in response to MTA feeding (Fig. 6a). In contrast, *R.* *rubrum* showed a more complicated response to MTA perturbation (Fig. 6b). Similar to *B.* *subtilis*, the purine salvage pathway following cleavage of the adenine moiety from MTA was also active. In addition, based on our observations, rubrum-type methionine salvage is linked to de novo purine biosynthesis, evidenced by an increase in the abundance of constituent metabolites observed after MTA feeding. In contrast to *B.* *subtilis*, the rubrum-type methionine salvage pathway is intertwined with isoprenoid biosynthesis (Erb et al. 2012). Notably, MTA feeding caused severe down-regulation of 1-deoxy-d-xylulose-5-phosphate synthase (Rru_A2619), the key enzyme of the non-mevalonate pathway that converts d-glyceraldehyde-3-phosphate into DXP (Eisenreich et al. 2004). Since this gene (Rru_A2619) was constitutively expressed regardless of sulfur sources (sulfate or MTA), both pathways (the rubrum-type methionine salvage and the non-mevalonate pathways) contributed to isoprenoid biosynthesis. However, upon MTA feeding, cells suppressed the influx from d-glyceraldehyde-3-phosphate (the non-mevalonate pathway), and activated the flux into DXP through the rubrum-type methionine salvage pathway in which glutathione plays a critical role as a coenzyme in vivo when 1 MTXu 5-P methylsulfurylase catalyzes dethiomethylation of MTXu 5-P. The rubrum-type methionine salvage pathway is also interconnected with the AMC (Hardie and Heurlier 2008; Doherty et al. 2010), in which re-methylation, a key reaction for recycling methionine from homocysteine, is unexpectedly suppressed. Since most bacteria synthesize methionine from homocysteine through re-methylation (Steegborn et al. 1999), and the *trans*-sulfuration pathway appears restricted to fungi and mammalian systems (Reveal and Paietta 2012), the repression of the re-methylation process in *R.* *rubrum* observed in our study was not anticipated. In contrast, genes in the purine salvage pathway, de novo purine biosynthesis and classical sulfur metabolism were not affected by MTA perturbation. This caused changes in the abundance of constituent metabolites without transcriptional regulation.

In Fig. 6b, we emphasize an unusual branch point in these coordinated pathways in which SAM is metabolized to SAH but not MTA. As accumulation of SAH can inhibit SAM-dependent methyltransferases; continuous depletion of SAH to homocysteine and adenosine is essential for maintaining normal methylation of DNA, RNA, proteins and other small molecules (Hoffman et al. 1980; James et al. 2002). In addition, it is known that up-regulated SAH can in turn up-regulate CBS and/or γ-cystathionase, and down-regulate 5,10-methylenetetrahydrofolate reductase (MTHFR). These regulatory functions can act in concert to reduce methionine re-methylation and expedite homocysteine removal in an attempt to normalize one-carbon flow (i.e., methyl group transfer). Homocysteine is catabolized to cysteine through cystathionine, and further metabolized into other important biological compounds such as pyruvate or glutathione, the latter being a reducing agent that protects the cell from oxidative stress. Since reducing equivalents in the form of reduced sulfur is necessary for the MTXu 5-P methylsulfurylase catalyzed dethiomethylation of MTXu 5-P, glutathione might be activated in vivo. Indeed, we detected the glutathione-methylthiol adduct via metabolomics, indicating that glutathione functions as a coenzyme in vivo in this process. In line with the metabolomics results, transcriptomics suggested that glutathione is tightly regulated to act in the metabolism of methylthiol released from MTXu 5-P. Glutathione is provided dynamically by NADPH-glutathione reductase from oxidized glutathione, and glutathione–methylthiol formation can be catalyzed by glutathione *S*-transferase (Rru_A0332). This adduct can be metabolized through the detoxification process by γ-glutamyl transpeptidase (Rru_A0385) and leucyl aminopeptidase (Rru_A0454), which were not induced but constitutively expressed in our RNAseq experiment. Also, cysteine from the *trans*-sulfuration can be catabolized into pyruvate metabolism and the TCA cycle, as evidenced by the RNAseq and metabolomics data.

To further investigate the actively changing metabolic pathways affected by glutathione and MTXu 5-P methylsulfurylase in *R.* *rubrum*, metabolite profiles of glutathione-related compounds in the wild-type organism were compared with those of an MTXu 5-P methylsulfurylase mutant (Rru_A2000, Fig. 5). Specifically, MTXu 5-P cleaved the pathway intermediate, methylthioxylulose-5-phosphate, into DXP and the methanethiol–glutathione adduct under the function of MTXu 5-P methylsulfurylase. In line with the critical function of MTXu 5-P methylsulfurylase, analysis of the MTXu 5-P methylsulfurylase mutant showed that only the purine salvage pathway was active, whereas other pathways in the mutant organism that are active in the wild type did not change relative to the control upon MTA-perturbation.

Interconnections identified using our approaches were also supported by metabolite–metabolite correlation analyses. Pearson correlation coefficients of percentage changes in metabolite abundances were clustered by agglomerative hierarchical clustering, clearly showing a high correlation between metabolites in each active pathway in *R.* *rubrum* (Supplementary Material Fig. S5). In addition, a strong correlation between interconnected active pathways could be observed; the rubrum-type methionine salvage pathway was strongly intertwined with the purine salvage pathway, de novo purine biosynthesis pathway, glutathione pathway, TCA cycle and the AMC. Also, there was a moderate correlation between the isoprenoid pathway and other active pathways (e.g., the methionine salvage, de novo purine biosynthesis, purine salvage, glutathione metabolism, butanoate metabolism, TCA cycle and the AMC). Metabolites in sulfur metabolism showed an anti-correlation with the other active pathways. When MTA was used as the sole sulfur source, no sulfate source could be provided through conventional sulfur metabolism; hence, the down-regulation of metabolites in that pathway was expected and mirrored the effect from transcriptomics. In *B.* *subtilis*, although metabolites in each pathway showed a strong correlation, there was only a weak correlation between the classical methionine salvage pathway and purine salvage pathway (Supplementary Material Fig. S6).

In addition, our analyses showed that there are two types of regulation, metabolic (i.e., changes in the metabolite levels) and hierarchical (i.e., transcription, translation and post-translational modification) (ter Kuile and Westerhoff 2001), that are induced by feeding MTA in *R.* *rubrum*. In *R.* *rubrum* the rubrum-type methionine salvage pathway, isoprenoid pathway, the AMC and the *trans*-sulfuration pathway were regulated hierarchically. In contrast, the purine salvage pathway, de novo purine biosynthesis pathway, and sulfur metabolism could be classified into metabolic regulation since corresponding genes were not induced by MTA feeding. Only the abundances of metabolites were changed upon MTA perturbation. In *B.* *subtilis* the subtilis-type methionine salvage pathway corresponded to hierarchical regulation. The purine salvage and de novo purine biosynthesis pathways were regulated only at the metabolite level.

These results show that our analytical strategy is useful for uncovering novel pathways, and that the addition of gene expression patterns provide complementary data to gain greater insight into bacterial metabolism. Therefore, untargeted metabolomics greatly aids the discovery of novel pathways. We expect our analytical pipeline to be applicable to many other systems and useful for uncovering a range of unexpected pathways.

## Electronic supplementary material

Below is the link to the electronic supplementary material.

Supplementary material 1 (PDF 978 kb)

Supplementary material 2 (XLS 1066 kb)

Supplementary material 3 (ZIP 23 kb)

## Abbreviations

AMC: Active methyl cycle
CBS: Cystathionine β-synthase
CGL: Cystathionine γ-lyase
DXP: 1-Deoxy-d-xylulose 5-phosphate
FT: Fourier transform
GSEA: Gene set enrichment analysis
HRPP: Hit ratio per peak
KEGG: Kyoto Encyclopedia of Gene and Genomes
LC: Liquid chromatography
MS: Mass spectrometry
MSEA: Metabolite set enrichment analysis
MTA: 5′-methylthioadenosine
MTHFR: 5,10-methylenetetrahydrofolate reductase
MTXu 5-P: 1-methylthio-d-xylulose 5-phosphate
qRT-PCR: Quantitative real time polymerase chain reaction
RNAseq: RNA sequencing
SAH: *S*-Adenosyl-l-homocysteine
SAM: *S*-Adenosyl-l-methionine
