# Supplementary material for: Integration of untargeted metabolomics with transcriptomics reveals active metabolic pathways
Source: Metabolomics. 2014 Aug 3;11(3):503–17. doi: 10.1007/s11306-014-0713-3 (PMC4334135; doi:10.1007/s11306-014-0713-3)
Supplement: Supplementary file 1 — Supplementary material 1 (PDF 978 kb) [file 11306_2014_713_MOESM1_ESM.pdf]

Integration of untargeted metabolomics with transcriptomics reveals active metabolic pathways

Kyuil Cho[†,‡] Bradley S. Evans, [†,‡] B. McKay Wood,[†] Ritesh Kumar,[†] Tobias J. Erb,[¶] Benjamin P. Warlick,[†,§] John A. Gerlt,[†,‡,§] and Jonathan V. Sweedler,[*,†,‡]

Electronic Supplementary Material

Table of Contents

Section 1. Supplementary Methods

1.1 Growth conditions and feeding experiment of *B. subtilis.*

1.2 Growth conditions and feeding experiment of *R. rubrum.*

1.3 *In vitro* reactions with 1-methylthio-D-xylulose-5-phosphate methylsulfurylase.

1.4 Liquid chromatography (LC)-Fourier transform (FT) mass spectrometry (MS) metabolomics.

1.5 LC-MS data analysis platform.

1.6 References.

Section 2. Supplementary Tables

Table S1. Performance comparison of putative peak annotation approaches for *R. rubrum.*

Table S2. Performance comparison of putative peak annotation approaches for *B. subtilis.*

Table S3. Actively changing metabolic pathways and their constituent metabolites in *B. subtilis* detected by the enrichment analysis.

Table S4. Abundance comparison of detected metabolites in the isoprenoid biosynthesis pathway between *R. rubrum* and *B. subtilis.*

Table S5. Actively changing metabolic pathways and their constituent metabolites of *R. rubrum* detected by the enrichment analysis.

Table S6. Genes and primers list used by qRT-PCR experiments.

Table S7. Quantitative real time polymerase chain reaction (qRT-PCR) of *B. subtilis.*

Table S8. Quantitative real time polymerase chain reaction (qRT-PCR) of *R. rubrum.*

Table S9. Genes and their CPMs of control (CPM_Ctrl) and treatment (CPM_Trt) in the RNAseq experiment (separate Excel file).

Section 3. Supplementary Figures

Figure S1. The LC–MS analysis platform system design.

Figure S2. Mass distribution of PubChem database.

Figure S3. Extracted ion chromatograms for glutathione-methylthiol disulfide for *in vitro* reaction.

Figure S4. Extracted ion chromatograms for glutathione-methylthiol disulfide for MTA feeding reaction.

Figure S5. Pearson correlation between metabolites in implicated pathways (*R. rubrum*).

Figure S6. Pearson correlation between metabolites in implicated pathways (*B. subtilis*).

Section 1. Supplementary Methods

1.1 Growth conditions and feeding experiment of *B. subtilis*

Cell growth: B. subtilis 168 (a gift from the laboratory of G. Ordal) was grown aerobically in either Luria Bertani broth (LB) or minimal medium at 37 °C with shaking at 220 rpm as previously described.(Sekowska and Danchin 2002) The minimal medium contained 8 mM K_2HPO_4, 4.4 mM KH_2PO_4, 27 mM glucose, 15 mM L-glutamine, 0.2 mM L-tryptophan, 2.48 µM $NaMoO_4$, 2.5 µM $CoCl_2$, 2.52 µM $CuCl_2$, 12.4 µM $ZnCl_2$, 5.05 µM $MnCl_2$, 50 µM $FeCl_3$, 49.5 µM $CaCl_2$, 610 µM $MgCl_2$, 33.5 µM iron citrate, and 300 µM trisodium citrate with either 0.5–1 mM Mg_2SO_4 or 0.5–1 mM MTA. Overnight LB cultures were washed with minimal medium lacking sulfur before 1:400 dilution into 200 mL minimal medium containing either sulfate or MTA as the sole sulfur source. The cultures were harvested by centrifugation at OD_{600} = 0.6 as measured by a Genesys 20 spectrophotometer (Thermo Scientific, Waltham, MA), and the cells were kept on ice unless otherwise indicated. The cells were washed with 50 mL minimal medium without sulfur before being resuspended in 5 mL of minimal medium without sulfur.

MTA feeding experiment: For three biological samples prepared as described above, metabolite depletion of the concentrated cells was immediately carried out at 37 °C in 5 mL minimal medium without sulfur for 10 min. The cells were then transferred back onto ice. The OD_{600} (*ca.* 24) was measured for each in triplicate by 50-fold dilution of a small aliquot into minimal medium in a cuvette. The averaged OD value of the concentrated cells was then used to dilute the cells to aliquots of OD_{600} = 6 at a final volume of 1 mL. Sulfur feeding was initiated by addition of concentrated MTA or sulfate to 1 mM before transferring to 37 °C for 0, 2, 5, or 15 min. Immediately after incubation, the cells were transferred to a 4 °C centrifuge and spun at 16,100 × g for 2 min, transferred to ice, and the supernatant was quickly removed by pipetting. Immediately following removal of the supernatant, the cells were frozen with liquid nitrogen and stored at –80 °C prior to extraction.

1.2 Growth conditions and feeding experiment of *R. rubrum*

Cell growth: R. rubrum (DSM 467, ATCC 11170) and its MTXu 5-P methylsulfurylase mutant (a gift from R. Tabita and Jaya Singh) were grown aerobically in LB medium or in the dark at 30° C on 20−2,000 mL minimal medium with sulfate or MTA as the sole sulfur source as described previously.(Erb et al. 2012) The minimal medium contained 17.9 mM malic acid, 35.0 mM NaOH, 22.4 mM NH_4Cl, 0.5 mM $CaCl_2 \times 2\ H_2O$, and 15 mM potassium phosphate buffer (pH 6.8), 1 mL of trace element solution (5 g EDTA disodium salt, 3 g $FeCl_2SO_4 \times 4H_2O$, 30 mg $MnCl_2 \times 4H_2O$, 50 mg $CoCl_2 \times 6H_2O$, 10 mg $CuCl_2 \times 2H_2O$, 20 mg $NiCl_2 \times 6H_2O$, 30 mg $Na_2MoO_4 \times 2H_2O$, 50 mg $ZnSO_4 \times 7H_2O$ and 20 mg H_3BO_3 per l, pH 3), and 1 mL of biotin solution (15 mg per L) or 1 mL of full vitamin solution (80 mg *p*-aminobenzoic acid, 100 mg calcium pantothenate, 100 mg cyanocobalamin, 20 mg biotin, 200 mg nicotinic acid, 300 mg pyridoxamine, 200 mg thiamine dichloride per L) with either 0.4–0.9 mM $MgSO_4 \times 7\ H_2O$ or 0.4−0.9 mM MTA and 0.4−0.9 mM $MgCl_2 \times 6\ H_2O$. The cultures were harvested at OD_{578} = 0.6 (Genesys 20 spectrophotometer) by centrifugation, and the cells were kept on ice unless otherwise indicated. The cells were then washed with 50 mL minimal media without sulfur.

1.3 *In vitro* reactions with 1-methylthio-D-xylulose-5-phosphate methylsulfurylase

Unless otherwise stated, chemicals used for synthesis were purchased from Sigma-Aldrich, and buffers and reaction mixture components were purchased from Fisher Scientific. Production of glutathione-methylthiol disulfide and DXP from MTXu 5-P was monitored by ^{1}H NMR with solvent suppression and LC-FTMS. The reactions (700 μL) in 100% H_2O contained: 60 mM Tris-HCl (pH 7.9), 15 mM $NaHCO_3$, 1 mM $MgCl_2$, 6 mM glutathione (or without glutathione), 4 mM MTXu/Ru 5-P, and 5 μM *R. rubrum* RLP. MTXu/Ru 5-P was enzymatically prepared from MTRu 1-P using the *R. rubrum* RLP. Immediately before inserting samples into the spectrometer, 100 μL of D_2O was added. The spectrum for MTXu/Ru 5-P was acquired and then the *R. rubrum* 1-methylthio-D-xylulose 5-phosphate methylsulfurylase was added to a final concentration of 10 μM.

1.4 Liquid chromatography (LC)-Fourier transform (FT) mass spectrometry (MS) metabolomics

Metabolite extraction: Metabolite extractions were performed at room temperature, with solvent B (10 mM ammonium bicarbonate buffer (pH 9.2) containing 90% (v/v) acetonitrile) used for the ZIC-pHILIC column (Merck SeQuant AB, S-907 19, Umeå, Sweden) on the LC-FTMS instrument described below. For each cell pellet originated from 1 mL of cells of OD_{600} = 6,375 μL, solvent B was added, vortexed for 15 min, and centrifuged two times at 16,100 × g for 5 min before adding to a fresh 96-well plate in the HPLC autosampler.

LC-FTMS analysis: LC-FTMS was performed using an 11T LTQ-FT Ultra mass spectrometer (Thermo Fisher Scientific, Waltham, MA) equipped with an Agilent 1200 HPLC system (Agilent Technologies, Santa Clara, CA). 100 μL of extracted metabolites were injected onto a 2.1 × 150 mm ZIC-HILIC column equilibrated with solvent A, 10 mM ammonium bicarbonate (pH 9.2). A linear gradient of 100% solvent B to 40% solvent B over 35 min, and then 40% solvent B to 100% solvent B in 10 min, followed by a 15 min re-equilibration at a flow rate 200 of μL/min was used for metabolomics experiments. For *in vitro* reaction monitoring, a shorter gradient was used: 100% B for 5 min followed by a linear gradient down to 40% B over 10 min, then a return to 100% B over 5 min followed by a 15 min re-equilibration for 15 min. In the case of *B. subtilis*, for each time point and condition (sulfate versus MTA feeding), three injections (technical replicates) were analyzed for each of three extractions (biological replicates). For *R. rubrum* analysis, four biological samples were each analyzed in triplicate for each time point and condition. All data were collected in negative profile mode at resolution of 50,000 with full scan set to *m/*z 100–1000.

1.5 LC-MS data analysis platform

Data processing: We used XCMS (Smith et al. 2006), a freely available and popular software package for LC-MS data pre-processing, including peak detection, peak matching, and retention time alignment. The raw LC-MS files were converted into XML format using the program MM File Conversion, one of the MassMatrix Mass spec Data File Conversion Tools (Xu and Freitas 2009). These XML-formatted files were used as input for XCMS. We used a binning method for fast data processing at the peak detection step, but it has some drawbacks. Apart from an optimal bin size issue, XCMS combines maximum signal intensities from adjacent slices into an overlapping combined chromatogram, thereby mixing potentially distinct peaks into a single peak. This peak mixing phenomenon reduces the benefits of using

high resolution LC-MS instruments, even though XCMS uses an intensity-weighted averaging strategy to reduce this effect. In addition, we might not detect close-by and partially overlapping features due to a post-processing step (implemented by the 'rectUnique()' function in the source code) that eliminates any peaks in the vicinity of higher intense peaks. This can be controlled by setting the 'mzdiff' option as a minus value, but in this case, we can get the same peak identification for several different peaks, which might be problematic for automatic data processing. XCMS provides another algorithm, centWave (Tautenhahn et al. 2008), that circumvents these issues by collecting the region of interest and using continuous wavelet transformation, but the longer processing time required by this method to search the entire list of regions of interest was prohibitive to our use. To properly address the issues with the binning method, we changed the source code in XCMS to filter minor mixing peaks in the major peaks using a 2 ppm mass tolerance in each bin allowing for more precise mass and area calculations. Also, we changed the rounding function to provide a precision of 5 decimal points for mass and to include retention times for peak identifications, allowing automatic processing in the following procedures. We set mzdiff = –1.001 to detect close-by and partially overlapping peaks. To get as many peaks as possible, we set the signal-to-noise threshold at 2 or to the lowest value, which does not produce peak alignment errors (*e.g.*, normally, snthresh = 2 or snthresh = 4). The parameters for xcmsSet(), group(), and rector() were set to the default values.

Data pre-processing: Multiple data pre-processing steps were introduced for data quality control (retention time filtering, non-biological peaks elimination, missing value imputation, and normalization), data redundancy elimination (adducts, isotopologues, and multimers), and automatic isotope pattern analysis for the experimental data. We eliminated peaks with retention times of less than 120 s or greater than 2,030 s, since salts and chemical impurities were frequently detected in these regions in our solvent system. Non-biological peaks were further filtered based on the mass distribution (*e.g.*, integral *versus* non-integral) of metabolites in the KEGG and PubChem databases (Figure 1 and Figure S2). The portion of peaks in between high density areas was just below 0.3%, even in the PubChem database, containing large numbers of synthetic molecules. Peaks in this area were eliminated by the following criteria: *i)* if integral ≤ 100, then non-integral ≥ 0.90 or ≤ 0.10; *ii)* if integral > 100 and ≤ 200, then non-integral ≥ 0.82 or ≤ 0.20; *iii)* if integral > 200 and ≤ 300, then non-integral ≥ 0.82 or ≤ 0.30; *iv)* if integral > 300 and ≤ 400, then non-integral ≥ 0.82 or ≤ 0.40; and *v)* if integral > 400 and ≤ 500, then non-integral ≥ 0.82 or ≤ 0.50). A non-biological filter was not applied when masses were greater than 500 *m/z* according to the mass distribution analysis. Missing intensity values were set to the integer values of the minimum intensity in each experimental data set (*i.e.*, missing value imputation). A matrix containing all-by-all mass differences was constructed to check adducts, multimers (*e.g.*, mostly dimers and trimers) and isotopologues (*e.g.*, C, N, S, and O) based on a 2 ppm mass tolerance and a 60-s retention time tolerance for all detected peaks. For every peak, if there were mass differences corresponding to adducts or multimers, those peaks were eliminated, and isotopologues were also removed after isotope patterns (*i.e.*, relative abundance and masses) were stored as a matrix for later use in molecular formula determination. For the remaining peaks, we calculated the mean intensities of biological and technical replicates and the relative ratios of mean intensities between control samples and treatment samples at each time point. Peaks were classified into the monoisotopic group or the isotopic group according to the presence of isotopologues. The isotopic

group was subdivided into the primary group and the secondary group based on the abundance change. The primary group included peaks with isotopic patterns showing > 20% of abundance change at each time point.

Molecular formula determination: For peaks within an isotopic group, molecular formulas were determined using six atoms (*e.g.*, C, N, O, P, S, and H), because most biological molecules are mainly composed of these atoms. Halogenated molecules were excluded at this point, but they can be easily included if necessary. Molecular formula determination was carried out using three functional modules: a theoretical molecular formula modeling module, theoretical isotopic pattern modeling module, and matching probability calculation module between theoretically predicted isotopic patterns and experimentally observed isotopic patterns. Theoretical molecular formulas were predicted based only on monoisotopic masses using previously published algorithms (Liptak 2007; Bocker et al. 2009; Bocker et al. 2008). Briefly, the algorithm constructs a data structure called an extended residue table to compute the smallest decomposable integer over given integer masses, and generates all possible decompositions of a query mass, recursively. For real-valued decompositions, the algorithm transforms the integer knapsack problem (Kellerer et al. 2004) with real-valued coefficients into a problem instance with integer coefficients by introducing a blowup factor (*i.e.*, scaling factor). This blowup factor inevitably causes a rounding error. To avoid this rounding error, previous studies decomposed additional integers by extending the upper bound using the maximum relative rounding error based on hydrogen atoms. A blowup factor can affect search space in the residue table due to the condition of coprimality between masses. In this study, we used 25,000 as a blowup factor. All of the possible molecular formulas were filtered by applying the seven golden rules (Kind and Fiehn 2007), except the trimethylsilylated compounds rule, which is only applicable to gas chromatography MS data, to select the most likely and chemically correct molecular formulas. For each predicted formula, we simulated theoretical isotopic patterns based on a dynamic programming approach in the context of the Markov process as described in a prior study (Snider 2007). We compared simulated isotopic patterns with the experimentally measured isotopic patterns based on Bayesian statistics, as described previously (W. Zhang and Chait 2000; N. Zhang et al. 2002; Bocker et al. 2009; J. Zhang et al. 2005). Only the top three ranked molecular formulas were used for searching against the KEGG database.

Seed metabolites: Peaks in the primary group were searched against the publicly available KEGG database, using nominal masses and the top three predicted molecular formulas with 5 ppm mass tolerance. If there were any hits, we considered those as seed metabolites, referring to the metabolites showing: higher abundance changes upon perturbation, clear isotope patterns in experimental data, and search hits in the database. Metabolic pathway information was extracted from these seed metabolites and used to construct initial pathway clusters. Since the same metabolite can participate in many different pathways, multiple pathway information can be extracted from a single seed metabolite. Initial pathway clusters were used to further annotate peaks in the secondary group and the monoisotopic group. Since it is difficult to annotate monoisotopic peaks with high confidence, even if there are hits in the database, additional information is needed to increase confidence. Retention time is starting to be used, but there are still some hurdles to overcome, such as different solvent systems. Pathway information and chemical reaction information can be used as alternatives to confer high confidence upon putative annotation.

Dynamic construction of metabolite sets (i.e. implicated pathways): After detecting seed metabolites and their respective pathways, peaks in the secondary group and the monoisotopic group were searched against the KEGG database with 5 ppm mass tolerance. Peaks in the secondary group can provide enhanced molecular formula information and increase the reliability of the search hits. For hits in the database, we compared the pathways and chemical reactions of those hits with those of the seed metabolites in the initial pathway clusters. If they matched with each other, we added those metabolites into the pathway clusters. This pathway comparison makes it possible to annotate monoisotopic peaks with high confidence which are usually difficult to annotate based solely on single mass information.

Metabolite set enrichment analysis (MSEA): Metabolite set enrichment analysis was carried out based on the same procedure and statistics as described in the previous gene set enrichment analysis (GSEA) studies (Subramanian et al. 2005; Mootha et al. 2003). After data processing, peaks were listed based on fold change. For each metabolite set, we calculated a maximum enrichment score by a normalized Kolmogorov-Smirnov (K-S) running sum statistic. For the metabolites $M_1, M_2, \cdots, M_n$ in the peak list and a metabolite set S containing N_h members, we used

$$X_i = -\sqrt{\frac{N_h}{(N - N_h)}}$$

if M_i is not a member of S, or

$$X_i = \sqrt{\frac{(N - N_h)}{N_h}}$$

if M_i is a member of S as the same way as the previous studies.

A running sum across all N metabolites was computed, and the maximum enrichment score (MES) was considered as the maximum observed positive deviation of the running sum for every metabolite set.

$$\text{MES} = \max_{1 \le j \le N} \sum_{i=1}^{j} X_i$$

For a null distribution, we permuted the class labels for 1,000 times and recorded the maximum enrichment score for every permutation. The maximum enrichment score from the actual data was then compared to this null distribution, providing a nominal P value to indicate statistical significance. Every metabolite set with $p < 0.05$ was considered as active metabolic pathway.

1.6 References

Bocker, S., Letzel, M. C., Liptak, Z., & Pervukhin, A. (2009). SIRIUS: decomposing isotope patterns for metabolite identification. *Bioinformatics, 25*, 218-224.

Bocker, S., Liptak, Z., Martin, M., Pervukhin, A., & Sudek, H. (2008). DECOMP--from interpreting Mass Spectrometry peaks to solving the Money Changing Problem. *Bioinformatics, 24*, 591-593.

Erb, T. J., Evans, B. S., Cho, K., Warlick, B. P., Sriram, J., Wood, B. M., et al. (2012). A RubisCO-like protein links SAM metabolism with isoprenoid biosynthesis. *Nat. Chem. Biol., 8*, 926-932.

Kellerer, H., Pferschy, U., & Pisinger, D. (2004). *Knapsack Problems*. Berlin Heidelberg: Springer.

Kind, T., & Fiehn, O. (2007). Seven Golden Rules for heuristic filtering of molecular formulas obtained by accurate mass spectrometry. *BMC Bioinformatics, 8*, 105.

Liptak, S. B. Z. (2007). A fast and simple algorithm for the Money Changing Problem. *Algorithmica, 48*, 19.

Mootha, V. K., Lindgren, C. M., Eriksson, K. F., Subramanian, A., Sihag, S., Lehar, J., et al. (2003). PGC-1alpha-responsive genes involved in oxidative phosphorylation are coordinately downregulated in human diabetes. *Nat. Genet., 34*, 267-273.

Sekowska, A., & Danchin, A. (2002). The methionine salvage pathway in Bacillus subtilis. *BMC Microbiol., 2*, 8.

Smith, C. A., Want, E. J., O'Maille, G., Abagyan, R., & Siuzdak, G. (2006). XCMS: processing mass spectrometry data for metabolite profiling using nonlinear peak alignment, matching, and identification. *Anal. Chem., 78*, 779-787.

Snider, R. K. (2007). Efficient calculation of exact mass isotopic distributions. *J. Am. Soc. Mass Spectrom., 18*, 1511-1515.

Subramanian, A., Tamayo, P., Mootha, V. K., Mukherjee, S., Ebert, B. L., Gillette, M. A., et al. (2005). Gene set enrichment analysis: a knowledge-based approach for interpreting genome-wide expression profiles. *Proc. Natl. Acad. Sci. U. S. A., 102*, 15545-15550.

Tautenhahn, R., Bottcher, C., & Neumann, S. (2008). Highly sensitive feature detection for high resolution LC/MS. *BMC Bioinformatics, 9*, 504.

Xu, H., & Freitas, M. A. (2009). Automated diagnosis of LC-MS/MS performance. *Bioinformatics, 25*, 1341-1343.

Zhang, J., Gao, W., Cai, J., He, S., Zeng, R., & Chen, R. (2005). Predicting molecular formulas of fragment ions with isotope patterns in tandem mass spectra. *IEEE/ACM Trans Comput Biol Bioinform, 2*, 217-230.

Zhang, N., Aebersold, R., & Schwikowski, B. (2002). ProbID: a probabilistic algorithm to identify peptides through sequence database searching using tandem mass spectral data. *Proteomics, 2*, 1406-1412.

Zhang, W., & Chait, B. T. (2000). ProFound: an expert system for protein identification using mass spectrometric peptide mapping information. *Anal. Chem., 72*, 2482-2489.

Section 2. Supplementary Tables

Table S1. Performance comparison of putative peak annotation approaches for *R. rubrum*.

	10 min			20 min			comment
	UID[a]	*TSH*[b]	*HRPP*[c]	*UID*[a]	*TSH*[b]	*HRPP*[c]	
Search by mass only (mass tolerance = 2 ppm)	147	416	***2.83***	151	450	***2.98***	Hard-to-annotate peaks putatively with mass information only.
Search by mass only (mass tolerance = 5 ppm)	228	705	***3.09***	261	779	***2.98***	Hard-to-annotate peaks putatively with mass information only.
Search with mass & molecular formula (mass tolerance = 5 ppm)	**41**	**109**	***2.66***	**34**	**84**	***2.47***	Molecular formula information can reduce false positives, but coverage also can be reduced.
Our seed metabolite approach	**120**	**271**	***2.26***	**72**	**148**	***2.05***	Seed metabolites and clustering by pathways can reduce false positives and increase coverage by using monoisotopic peaks without isotopic information.

[a]UID: Number of unique peak identifications having search hits in KEGG database.
[b]TSH: Total number of search hits in KEGG database.
[c]HRPP: Hit ratio per peak calculated by TSH/UID.

The performance of putative peak annotation is represented by the hit ratio per peak (HRPP). Note that our approach shows the lowest HRPP values but the higher number of unique peaks in comparison with the conventional approach *(i.e.*, search with mass and molecular formulas), implying better coverage and lower false positives. Search based only on mass cannot annotate peaks with confidence without any biological knowledge and manual curation.

Table S2. Performance comparison of putative peak annotation approaches for *B. subtilis*.

	**2 min**			**5 min**			**15 min**			comment
	UID[a]	*TSH*[b]	*HRPP*[c]	*UID*[a]	*TSH*[b]	*HR*[c]	*UID*[a]	*TSH*[b]	*HRPP*[c]	
Search by mass only (mass tolerance = 2ppm)	136	337	***2.48***	111	278	***2.50***	119	260	***2.18***	Hard to annotate peaks putatively with mass information only.
Search by mass only (mass tolerance = 5ppm)	291	749	***2.57***	288	717	***2.49***	260	641	***2.47***	Hard to annotate peaks putatively with mass information only.
Search with mass & molecular formula (mass tolerance = 5ppm)	**33**	**93**	***2.82***	**23**	**65**	***2.83***	**19**	**50**	***2.63***	Molecular formula information can reduce false positives, but coverage also can be largely reduced.
Our seed metabolite approach	**105**	**198**	***1.89***	**106**	**214**	***2.02***	**78**	**154**	***1.97***	Seed metabolites and clustering by pathways can reduce false positives, and increase coverage by using monoisotopic peaks without isotopic information.

[a]UID: Number of unique peak ID having search hits in KEGG database
[b]TSH: Total number of search hits in KEGG database
[c]HRPP: Hit ratio per peak calculated by TSH/UID

A similar pattern of performance was observed in the *B. subtilis* metabolomics data. Our approach shows better coverage and lower false positives.

Table S3. Actively changing metabolic pathways and their constituent metabolites in B. subtilis detected by the enrichment analysis.

Metabolites			Fold change			p-value			Pathway
Name	Monoisotopic mass (*m/z*)	Retention time (*sec*)	2 min	5 min	15 min	2 min	5 min	15 min	
Adenine	134.0471	244	4.66	7.41	8.96	8.43×10^{-4}	1.57×10^{-3}	4.19×10^{-3}	Purine salvage
Hypoxanthine	135.0304	309	6.93	6.15	20.18	0.02	0.03	2.18×10^{-3}	Purine salvage
Deoxyadenosine	250.0944	205	2.57	3.49	1.55	0.06	6.79×10^{-3}	0.35	Purine salvage
Deoxyinosine	251.0785	274	9.68	2.80	-	0.048	0.28	-	Purine salvage
Adenosine/ Deoxyguanosine	266.0894	235	1.03	1.32	1.16	0.87	0.30	0.50	Purine salvage
Inosine	267.0738	435	2.47	1.42	0.97	0.16	0.52	0.96	Purine salvage
Guanosine	282.0838	881	0.56	-	-	0.49	-	-	Purine salvage
Xanthosine	283.0686	1486	0.45	0.08	-	0.24	0.30	-	*De novo* purine biosynthesis
AICAR	337.0543	1507	1.02	0.99	1.29	0.97	0.99	0.73	*De novo* purine biosynthesis
AMP/dGMP/3'-AMP	346.0555	1534	1.22	1.16	0.01	0.71	0.45	0.12	*De novo* purine biosynthesis
Xanthosine-5'-phosphate	363.0346	1566	0.50	0.35	0.36	2.21×10^{-3}	3.53×10^{-3}	8.77×10^{-3}	*De novo* purine biosynthesis
MTP[a]	119.0171	191	91.35	1.17	-	2.74×10^{-4}	0.46	-	met salvage pathway
MTOB[b]	147.0120	172	34.70	-	-	1.13×10^{-4}	-	-	met salvage pathway
L-Methionine	148.0437	559	2.91	50.00	-	7.30×10^{-3}	0.02	-	met salvage pathway
DHK-MTPene[c]	161.0285	201	11.48	4.64	5.08	6.54×10^{-4}	1.98×10^{-3}	0.02	met salvage pathway
MTR	179.0383	190	3976.13	3927.10	1421.71	1.16×10^{-4}	1.79×10^{-4}	2.53×10^{-3}	met salvage pathway
DK-MTP-1P/ HK-MTPenyl-1P[d]	240.9939	203	1.05	-	-	0.80	-	-	met salvage pathway
MTR-1P/MTRu-1P/MTXu-1P[e]	259.0046	1514	14.1	357.42	93.44	5.76×10^{-4}	2.04×10^{-5}	1.12×10^{-3}	met salvage pathway
MTA[f]	296.0825	181	40.17	32.18	95.48	4.63×10^{-3}	1.33×10^{-3}	6.56×10^{-4}	met salvage pathway

[a] 3'-Methylthiopropionate
[b] 4-Methylthio-2-oxobutanoate
[c] 1,2-Dihydroxy-3-keto-5-methylthiopentene

[d] 2,3-Diketo-5-methylthiopentyl-1-phosphate/2-Hydroxy-3-keto-5-methylthiopentenyl-1-phosphate
[e] S-Methyl-5-thio-D-ribose/S-Methyl-5-thio-D-ribose-1-phosphate/S-Methyl-5-thio-D-ribulose-1-phosphate/S-Methyl-5-thio-D-xylulose-1-phosphate
[f] 5'-Methylthioadenosine

The subtilis-type methionine salvage pathway and the purine salvage pathway were detected as active pathways based on the MSEA. Fold changes and their corresponding p-values calculated by ANOVA were represented at selected time points. Metabolites in the subtilis-type methionine salvage pathway and the purine salvage pathway were up-regulated, and some of the metabolites in *de novo* purine biosynthesis were down-regulated or not affected upon MTA feeding at the metabolite levels.

Table S4. Abundance comparison of detected metabolites in the isoprenoid biosynthesis pathway between *R. rubrum* and *B. subtilis*.

(a) *Rhodospirillum rubrum*

Metabolites			Fold change		p-value		Pathway
Name	Monoisotopic mass (*m/z*)	Retention time (sec)	10 min	20 min	10 min	20 min	
DXP	213.0166	1497	5.55	417.77	2.67×10^{-4}	1.24×10^{-3}	Isoprenoid biosynthesis
c-MEP	276.9878	1576	7.12	9.98	4.63×10^{-11}	3.86×10^{-10}	Isoprenoid biosynthesis
CDP-ME	520.0728	1652	221.70	29.93	3.50×10^{-6}	7.25×10^{-9}	Isoprenoid biosynthesis

(b) *Bacillus subtilis*

Metabolites			Fold change			p-value			Pathway
Name	Monoisotopic mass (*m/z*)	Retention time (sec)	2 min	5 min	15 min	2 min	5 min	15 min	
MEP	215.0327	1513	0.90	0.88	1.17	0.65	0.53	0.63	Isoprenoid biosynthesis
c-MEP	276.9880	1552	1.01	1.26	1.76	0.92	0.11	0.04	Isoprenoid biosynthesis
CDP-ME	520.0738	1550	0.97	0.87	1.32	0.62	0.26	0.12	Isoprenoid biosynthesis

Metabolites in isoprenoid biosynthesis are highly up-regulated in *R. rubrum*, but they are not affected in *B. subtilis* upon MTA perturbation.

Table S5. Actively changing metabolic pathways and their constituent metabolites in *R. rubrum* detected by the enrichment analysis.

Metabolites			***Fold change***		***p-value***		**Pathway**
Name	Monoisotopic mass (*m/z*)	Retention time (sec)	10 min	20 min	10 min	20 min	
Adenine	134.0467	180	75.35	64.94	4.78×10^{-17}	1.63×10^{-12}	Purine salvage
Hypoxanthine	135.0307	566	-	4.62	-	1.34×10^{-6}	Purine salvage
Guanine	150.0416	128	-	1.51	-	0.07	Purine salvage
Urate	167.0205	152	-	3.44	-	3.54×10^{-3}	Purine salvage
Adenosine/ Deoxyguanoisine	266.0890	394	-	0.64	-	0.11	Purine salvage
Guanosine	282.0839	1102	3.6	1.09	5.85×10^{-6}	0.43	Purine salvage
dIMP	331.0479	1504	88.99	72.67	5.61×10^{-14}	1.07×10^{-3}	Purine salvage
dAMP	330.0604	1382	35.51	0.77	1.27×10^{-8}	0.14	Purine salvage
N-formyl-GAR	313.0437	1614	244.27	-	2.81×10^{-13}	-	*De novo* purine biosynthesis
AMP/3’-AMP/dGMP	346.0551	1502	263.93	73.11	5.00×10^{-10}	3.54×10^{-3}	*De novo* purine biosynthesis
GMP/3’-GMP	362.0500	1660	50.82	1.04	1.46×10^{-9}	0.73	*De novo* purine biosynthesis
cGMP	344.0396	1262	-	0.58	-	0.32	*De novo* purine biosynthesis
ADP/PAP/dGDP	426.0213	1723	-	0.24	-	1.33×10^{-3}	*De novo* purine biosynthesis
GTP	521.9828	1015	-	0.47	-	0.02	*De novo* purine biosynthesis
ADP-ribose	558.0634	1533	636.60	2.03	1.14×10^{-11}	4.39×10^{-4}	*De novo* purine biosynthesis
MTA	296.0818	186	206.86	104.35	9.91×10^{-7}	1.33×10^{-10}	*Rubrum-type* met salvage
MTR	179.0381	183	16.43	81.65	5.59×10^{-6}	1.68×10^{-9}	*Rubrum-type* met salvage

MTR-1P/MTRu-5P/MTXu-5P	259.0042	1373	1647.16	4997.37	5.36×10^{-13}	1.24×10^{-3}	*Rubrum-type* met salvage
MTRu-1P	259.0042	1435	565.65	852.11	1.00×10^{-10}	1.22×10^{-3}	*Rubrum-type* met salvage
DXP	213.0166	1497	5.55	417.77	2.67×10^{-4}	1.24×10^{-3}	Isoprenoid biosynthesis
c-MEP	276.9878	1576	7.12	9.98	4.63×10^{-11}	3.86×10^{-10}	Isoprenoid biosynthesis
CDP-ME	520.0728	1652	221.70	29.93	3.50×10^{-6}	7.25×10^{-9}	Isoprenoid biosynthesis
S-Adenosyl-L-homocysteine	383.1135	1385	702.93	0.65	4.72×10^{-14}	5.02×10^{-4}	Active methyl cycle
S-Adenosyl-L-methione	397.1291	1588	148.43	0.47	6.22×10^{-7}	2.12×10^{-4}	Active methyl cycle
O-Acetyl-L-serine	146.0456	1476	0.0005	0.64	2.55×10^{-4}	0.02	Sulfur metabolism
O-Acetyl-L-homoserine	160.0613	427	-	0.20	-	1.53×10^{-3}	Sulfur metabolism
Adenosine-3’,5’-bisphosphate	426.0215	1723	-	0.49	-	1.33×10^{-3}	Sulfur metabolism
5’-Oxoproline	128.0351	1519	0.65	-	0.25	-	Glutathione metabolism
Glutamate	146.0456	1523	28.10	0.64	8.03×10^{-4}	0.02	Glutathione metabolism
Ascorbate	175.0246	341	33.92	0.20	3.78×10^{-5}	1.46×10^{-5}	Glutathione metabolism
Glutathione	306.0759	1525	510.45	0.0034	0.05	0.08	Glutathione metabolism
Methylthiolglutathione	352.0636	1338	914.34	1573.83	1.35×10^{-11}	1.05×10^{-4}	Glutathione metabolism
S-Glutathionyl-L-cysteine	425.0797	1758	75.55	1.92	9.53×10^{-6}	0.10	Glutathione metabolism
Glutathione disulfide	611.1442	1816	1.31	0.65	0.02	0.09	Glutathione metabolism

Fumarate	115.0035	1610	7.94	1.0	4.59×10^{-12}	0.02	TCA cycle
Succinate	117.0191	1561	0.31	0.74	6.29×10^{-3}	9.20×10^{-6}	TCA cycle
Malate	133.0140	1616	10.96	1.01	2.20×10^{-10}	0.04	TCA cycle
2-Oxoglutarate	145.0140	1566	269.65	74.42	1.91×10^{-12}	1.04×10^{-3}	TCA cycle
α-ketobutyrate	101.0242	348	0.048	0.47	6.54×10^{-5}	0.04	Butanoate metabolism
Hydroxybutanoate	103.0396	518	1.59	1.91	0.37	0.30	Butanoate metabolism
2-Acetolactate	131.0348	191	1.89	0.38	0.01	3.32×10^{-11}	Butanoate metabolism
Glutamate	146.0456	1523	28.1	0.64	8.03×10^{-4}	0.02	Butanoate metabolism
2-Hydroxyglutarate	147.0296	1573	96.89	0.80	8.29×10^{-15}	3.05×10^{-3}	Butanoate metabolism

In total, eight pathways were detected as active pathways by MSEA. Fold changes and their corresponding p-values by ANOVA are represented at selected time points. Except for sulfur metabolism, detected pathways are up-regulated at the metabolite levels.

Table S6. Genes and primers list used by qRT-PCR experiments.

Genes	Primers
B. subtilis	
16SBsF	AGTGATGAAGGTTTTCGGAT
16SBsR	GCTCCTCAGCGTCAGTTACA
BSU13620F	GGGATATCACGAGGCTTC
BSU13620R	CAGGGTTTAAACGGACATCAA
BSU27270F	GGAAAAGTAAATGCTGCTAT
BSU27270R	ATCGCCTGTTGCAATCGTTC
BSU13560F	GAATCGAAAGCAGCGCTCTC
BSU13560R	AAACAAGTCTTTCCGTAATG
BSU13550F	TCATGGGCGCTTGAGAGACT
BSU13550R	ACCTTGCAGGACAGGCCTTG
BSU16550F	TAGAAGCAGCAGTGATGGAG
BSU16550R	CCCGCGTCTTGTCCCGAAAA
BSU00090F	TCAGAAAGCCCTGGTGAAAC
BSU00090R	CATGCCGCGGTATACCTTA
BSU06440F	GTGACCGCCATGCTGACTA
BSU06440R	GCGAATTTCTCGATGCTTGT
BSU14520F	CGCTGAATATCCATTTTATGCTT
BSU14520R	CCTGATCGCTCAAAACTTGC
BSU27610F	GCTTGGCGTAGGTTTTGC
BSU27610R	GCCATAATCCACTTTGATTACTTCA
BSU00140F	TAAACGGCTTGGATTTAAAACAT
BSU00140R	TGAAAGCTCCAACGTTCAAA
BSU07840F	GTCAAGGACTGGCTGGAGAT
BSU07840R	CGCCTTTAGCTGGATCAATTT
BSU23490F	CGGAAGTCATTGTAGCGAATC
BSU23490R	CGCGTTAGAGATGCAGGAA
BSU19630F	TCACCGTGAGTGATCACGTATT
BSU19630R	TGTCGTTTGACGCTCTTCC
BSU27610F	GCTTGGCGTAGGTTTTGC
BSU27610R	GCCATAATCCACTTTGATTACTTCA
BSU00680F	TGATGAAACATGATATCGAGAAAGT
BSU00680R	CGCTCGTTAATTCTGCACCTA
BSU06520F	AACAGCTGACATCCGTTCAA
BSU06520R	TCGAAGCCGTGCATATCTAA
BSU24270F	TGACTTTGTAAAGCCGAAAGC
BSU24270R	ACAGTTCCGCTGACAAGACC

Genes	Primers
R. rubrum	
Rr16SRTF	GTAACACCAGAAGAAGCCCC
Rr16SRTR	CATGTACCCCGACATCTAGCA
Rru_A1692F	CCCATATCAGCATCCACACA
Rru_A1692R	GTCGCCGCACATGAAGAT
Rru_A2483F	GCCTTCTTCATCTCAACGACA
Rru_A2483R	CTTCATCTCGGCCAATCC
Rru_A0149F	TCGAACGGCGAATTCCTA
Rru_A0149R	GCTGGCGACTCTGTCTGG
Rru_A2168F	GGCGCCGATATCATCTGT
Rru_A2168R	GATGTTGATCATCCTGTCTTGC
Rru_A1963F	GCATGAGCGCGATATGAG
Rru_A1963R	CAAGGACAAGGGTGGTATCG
Rru_A3655F	AGACCCTGCATCCGAAGAT
Rru_A3655R	CTGGTGCTCGGGATTGTC
Rru_A0917F	CTTCGGAACGGAGACCATC
Rru_A0917R	GAAACCGAGAAGGGAAAGGT
Rru_A1998F	ATGCCGGTCAGCTTTTCA
Rru_A1998R	ATCAAGCAAGACGGTGTCGT
Rru_A2289F	TATTCGATCGGCAAGGACA
Rru_A2289R	CGAGGGATAGAACGCCTTG
Rru_A0774F	TAACCTTTCCCGGGTTGC
Rru_A0774R	ATAGCCGCCCTTGAGATAGG
Rru_A0098F	CAGGTGATCTCCTATGCGGTA
Rru_A0098R	GTCGGGATGTAGAACAACAGG
Rru_A1616F	GGTGGAACCCCGCTTATT
Rru_A1616R	AACTCGGCCTTGCCTAAAAT
Rru_A0263RTF	GTCGCGCTTGTGGAAGAT
Rru_A0263RTR	GCGCAAGACCTCGTCGATCA
Rru_A1592RTF	CTGACCGCCAATGGCAATGC
Rru_A1592RTR	GGCGTTGTGTTCGGAATCAA
Rru_A0054RTF	TCGAAAAGCTGGCTGTCGAT
Rru_A0054RTR	CTGCGGCTTGGCCTTTTCCT
Rru_A0774RTF	ATCGGCTTCGCCTATACCCG
Rru_A0774RTR	GGCGGCGACCTCGGCGAT
Rru_A0361RTF	CGTGGCCATGTGCTCGC
Rru_A0361RTR	ATAGAGATTGCTTTCGG
Rru_A360RTF	CCGATCAATATCCTGACCCA
Rru_A360RTR	CAGCGCCACATAGAAGGGCA
Rru_A2000RTF	CGTCGATCTGCTCGCCTAT

Genes	Primers
Rru_A2000RTR	CATCGCCAGATCGGCCTCGG
Rru_A0607RTF	TACGATATCTCGACGTTGCTG
Rru_A0607RTR	ATTGGCGCCCATCTTGCCAA
Rru_A0299RTF	GTCGCCCGCTTCGCCGTGAT
Rru_A0299RTR	GCGGGCCCCCGGCGGCACGA
Rru_A1057RTF	ATCGAGGATATCCGCAAGGA
Rru_A1057RTR	CCCGTGCTTGGCCGCCACAT
Rru_A0787RTF	GAGCCGGGAAAAGATCGCC
Rru_A0787RTR	CCCGATGTCACGTAATCGGCC
Rru_A0786RTF	CAATCCGACCCGCGCCGCCT
Rru_A0786RTR	GTGCGCAGGCCATGCTCCTT
Rru_A1530RTF	CAAGGGCTTGCGCGACGTCGC
Rru_A1530RTR	AGGGTCGCCGCCACCATGCG
Rru_A1531RTF	GTTCGACATGCTCAAGACCT
Rru_A1531RTR	TTGTGCACATATTCGGCCAT
Rru_A1682RTF	GGTGGTTCGCCGCAACCGCGT
Rru_A1682RTR	GTGACCAGGGCGCCATCCTTG
Rru_A1254RTF	GACCGACCCCGAGGCCTTCG
Rru_A1254RTR	CCGCCATTGATCAACTCGTC
Rru_A1691RTF	AACCGTCGAGCGGGCGACCA
Rru_A1691RTR	TAGAAGGCCACCTCGGGGAA
Rru_A2619RTF	CAAACCCTTCGACGAGGA
Rru_A2619RTR	CTCGACGACGATCAGCAC
Rru_A0784RTF	TTCGACGACCAAATACATCG
Rru_A0784RTR	TGCCGCTATCGATGATGAC
Rru_A3776RTF	CGACAGCCTGACTCACGA
Rru_A3776RTR	CATAGCCGATATCCTTGATCG
Rru_A1836RTF	CTGGTCGGCTTTGAACTGA
Rru_A1836RTR	TTCAGGGCGTCGATGAAG
Rru_A2075RTF	CGTCGATGTCGAACAGGTC
Rru_A2075RTR	GTCGACGATCAGCGGAAT

Table S7. Quantitative real time polymerase chain reaction (qRT-PCR) of *B. subtilis.*

Genes	Fold change				Function	Pathway
	0 min	2 min	5 min	15 min		
BSU27270	1.2 (0.2)*	4.1 (0.2)	5.9 (0.2)	2.9 (0.2)	5'-Methylthioadenosine/S-adenosylhomocysteine nucleosidase	Subtilis-type met-salvage
BSU13560	1.5 (0.4)	5.6 (0.4)	4.4 (0.2)	2.9 (0.3)	Methylthioribose kinase	
BSU13550	1.5 (0.3)	7.9 (0.4)	9.5 (0.4)	3.2 (0.2)	Methylthioribose-1-phosphate isomerase	
BSU13620	1.1 (0.1)	3.5 (0.3)	5.4 (0.2)	3.1 (0.2)	Acireductone dioxygenase	
BSU24270	1.2 (0.1)	1.1 (0.1)	1.4 (0.3)	1.3 (0.2)	1-Deoxy-D-xylulose 5-phosphate synthase	Non-mevalonate isoprenoid biosynthesis
BSU16550	1.1 (0.1)	-1.6 (0.1)	-2.9 (0.1)	-1.6 (0.2)	1-Deoxy-D-xylulose 5-phosphate reductoisomerase	
BSU14520	1.3 (0.3)	-1.1 (0.1)	-1.1 (0.2)	1.1 (0.2)	Adenine deaminase	Purine salvage
BSU27610	1.3 (0.2)	1.3 (0.3)	1.9 (0.4)	1.9 (0.2)	Adenine phosphoribosyltransferase	
BSU00680	1.1 (0.1)	1.5 (0.2)	1.5 (0.2)	1.9 (0.3)	Hypoxanthine-guanine phosphoribosyltransferase	
BSU23490	1.3 (0.1)	1.9 (0.1)	1.5 (0.2)	1.1 (0.1)	Purine nucleoside phosphorylase	
BSU19630	1.4 (0.3)	1.6 (0.2)	1.9 (0.2)	1.4 (0.3)	Purine nucleoside phosphorylase	
BSU07840	1.6 (0.2)	1.3 (0.2)	1.7 (0.2)	1.9 (0.2)	Bifunctional 2',3'-cyclic nucleotide 2'-phosphodiesterase	
BSU00140	1.7 (0.2)	1.6 (0.2)	1.2 (0.1)	1.7 (0.2)	Deoxyadenosine/deoxycytidine kinase	
BSU06440	1.6 (0.2)	1.3 (0.2)	1.7 (0.2)	-1.1 (0.1)	Adenylosuccinage lyase	*De novo* purine biosynthesis
BSU06520	1.4 (0.2)	1.8 (0.2)	1.9 (0.3)	1.5 (0.2)	Bifunctional phosphoribosylaminoimidazolecarboxamide	
BSU00090	1.4 (0.3)	1.1 (0.1)	1.5 (0.2)	1.2 (0.2)	Inosine 5'-monophosphate dehydrogenase	

*( ) stands for standard deviation

Candidate genes for qRT-PCR are manually selected based on metabolomics analysis. The purine metabolism is divided into the purine salvage pathway and the *de novo* purine biosynthesis pathway. Primer sequences read 5' to 3', and expression patterns are monitored at selected time points (0 min, 2 min, 5 min, and 15 min).

Table S8. Quantitative real time polymerase chain reaction (qRT-PCR) of *R. rubrum*.

Genes	Fold change 0 min	10 min	20 min	0.6 OD	Function	Pathway
Rru_A0361	1.6 (0.2)*	5.5 (0.5)	6.9 (0.6)	1.1 (0.2)	5'-Methylthioadeonsine phosphorylase	Rubrum-type met-salvage
Rru_A0360	1.1 (0.1)	4.5 (0.4)	4.9 (0.3)	1.2 (0.2)	Methylthioribose-1-phosphate isomerase	
Rru_A1998	1.0 (0.1)	3.4 (0.4)	4.3 (0.3)	1.1 (0.1)	Ribulose 1,5-bisphosphate carboxylase	
Rru_A2000	1.1 (0.1)	2.9 (0.4)	3.1 (0.4)	1.2 (0.2)	MTXu 5-P methylsulfurylase	
Rru_A0774	1.1 (0.1)	6.7 (0.2)	7.9 (0.5)	3.5 (0.4)	O-acetylhomoserine/O-acetylserine sulfhydrylase	
Rru_A0784	1.1 (0.1)	8.1 (0.2)	5.7 (0.5)	7.2 (0.7)	O-acetylhomoserine/O-acetylserine sulfhydrylase	
Rru_A2619	-1.1 (0.2)	-3.8 (0.4)	-8.8 (0.7)	1.2 (0.1)	1-Deoxy-D-xylulose-5-phosphate synthase	Non-mevalonate
Rru_A1592	1.1 (0.1)	4.9 (0.2)	7.4 (0.7)	1.4 (0.4)	1-Deoxy-D-xylulose 5-phosphate reductoisomerase	Isoprenoid biosynthesis
Rru_A0263	-1.1 (0.1)	6.6 (0.3)	7.9 (0.6)	1.5 (0.2)	4-Diphosphocytidyl-2-C-methyl-D-erythritol kinase	
Rru_A3776	-1.3 (0.1)	2.1 (0.2)	3.1 (0.2)	-1.2 (0.2)	S-adenosylmethionine synthetase	Active methyl cycle
Rru_A1531	1.1 (0.1)	-5.1 (0.3)	-4.9 (0.3)	-1.2 (0.2)	Methionine synthase	
Rru_A1530	1.6 (0.2)	-4.5 (0.4)	-3.9 (0.3)	-1.2 (0.2)	5,10-Methylenetetrahydrofolate reductase	
Rru_A0787	1.1 (0.1)	6.7 (0.6)	7.9 (0.3)	4.5 (0.3)	Cystathionine β-synthase	Trans-sulfuration
Rru_A0786	1.1 (0.1)	5.8 (0.2)	8.2 (0.5)	5.1 (0.3)	Cystathionine γ-lyase	
Rru_A1691	1.1 (0.1)	1.4 (0.2)	1.2 (0.2)	-1.2 (0.2)	Spermidine synthase	Arginine metabolism
Rru_A1254	1.3 (0.1)	-1.1 (0.1)	-1.4 (0.1)	1.1 (0.4)	Putrescine aminotransferase	
Rru_A1692	1.1 (0.2)	-1.1 (0.1)	1.1 (0.1)	-1.5 (0.2)	S-adenosylmethionine decarboxylase related	
Rru_A0149	1.2 (0.1)	1.3 (0.1)	-1.1 (0.1)	1.3 (0.1)	PfkB protein	Purine salvage
Rru_A2483	1.1 (0.2)	1.3 (0.2)	1.2 (0.1)	1.5 (0.2)	5'-Nucleotidase	
Rru_A0607	1.6 (0.1)	1.1 (0.1)	1.2 (0.2)	1.4 (0.2)	Adenine phosphoribosyltransferase	
Rru_A2168	1.3 (0.3)	-1.1 (0.2)	1.1 (0.2)	1.4 (0.2)	Phosphoribosylglycinamide formyltrasferase	*De novo* purine biosynthesis
Rru_A1963	1.3 (0.2)	1.1 (0.2)	1.2 (0.2)	-1.3 (0.1)	Adenylosuccinate lyase	
Rru_A0299	1.1 (0.2)	1.2 (0.1)	1.3 (0.3)	1.1 (0.2)	Glutamine amidotransferase	
Rru_A3655	1.3 (0.2)	1.3 (0.2)	1.1 (0.2)	1.2 (0.1)	Phosphoribosylaminoimidazolecarboxamide formyltranferase	
Rru_A2289	-1.1 (0.2)	-1.7 (0.3)	-2.1 (0.2)	-3.2 (0.2)	Sulfate adenylyltransferase subunit 2	Sulfur metabolism
Rru_A0098	1.0 (0.1)	1.2 (0.1)	1.3 (0.2)	1.1 (0.2)	Cystathionine γ-synthase	
Rru_A1616	1.4 (0.2)	1.1 (0.1)	-1.1 (0.2)	1.5 (0.2)	Cysteine synthase A	

*( ) stands for standard deviation

Candidate genes for qRT-PCR are selected manually based on metabolomics analysis. Detected active pathways are further categorized based on previous studies and plausible hypothesis. Primer sequences read 5' to 3', and expression patterns are monitored according to the time points (*i.e.*, 0 min, 10 min, 20 min, 0.6 OD).

Table S9. Genes and their counts per million (CPM) of control (CPM_Ctrl) and treatment (CPM_Trt) in the RNAseq experiment for *R. rubrum*.

See separate Excel file titled Supplementary Material 2 (Table S9).

Section 3. Supplementary Figures

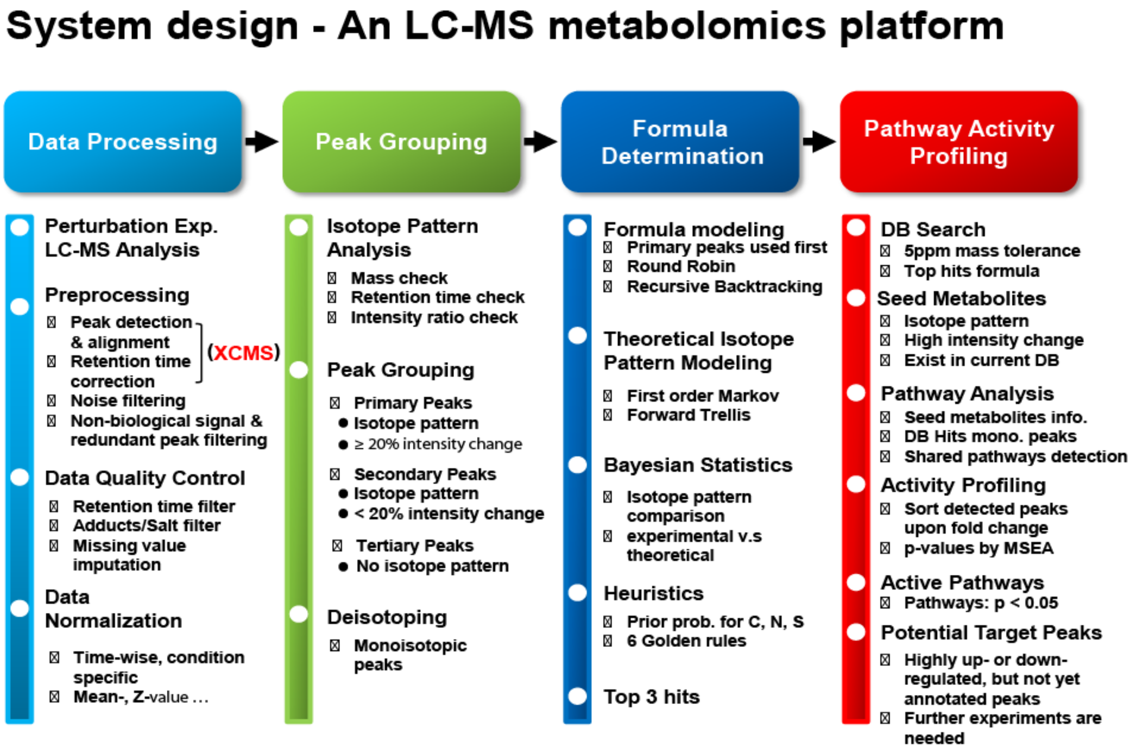

Fig. S1. The LC-MS analysis platform system design. Our LC-MS analysis platform was composed of four major functional modules, including data processing, peak grouping, molecular formula determination and pathway activity profiling modules. Each module consisted of sub-modules to deal with arising issues in the metabolomics data analysis. Non-biological signals and redundant peaks were filtered by a data pre-processing sub-module. Missing values were imputed at the data quality control stage. Remaining peaks were categorized into the isotopic group (primary and secondary groups) or the tertiary group. Molecular formulas were predicted in the molecular formula prediction module based on previous published algorithms. Based on predicted molecular formulas and nominal masses, peaks were putatively annotated and active pathways were assigned by MSEA. Notably, the concept of seed metabolites and dynamic build-up of metabolite sets to be evaluated enabled the streamlined process to detect active pathways from raw LC-MS data. These detected active pathways were further validated by transcriptomics, and metabolites highly perturbed but not annotated were listed for further experiments.

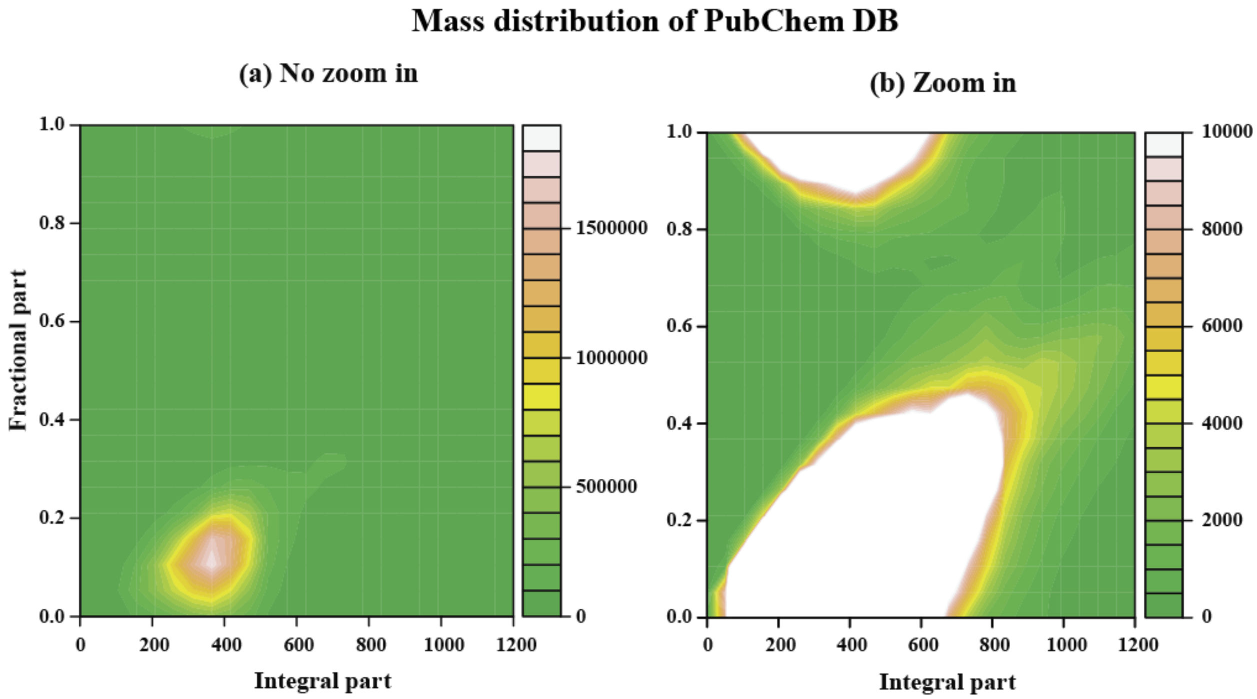

Fig. S2. Mass distribution of PubChem database. The distribution of the integral parts and the non-integral parts of the masses in the PubChem database was investigated as a proof-of-concept of the mass filter. As with the KEGG database, there was a region not occupied by molecules in PubChem, even though many synthetic molecules are listed in the PubChem database. Only > 0.3% of molecules appeared in-between the high density areas, indicating that this region can be used as a mass filter for non-biological signals at the data processing step without much information loss. By eliminating spurious peaks in this region, we enhanced processing time and concentrated on the major peaks in the subsequent steps.

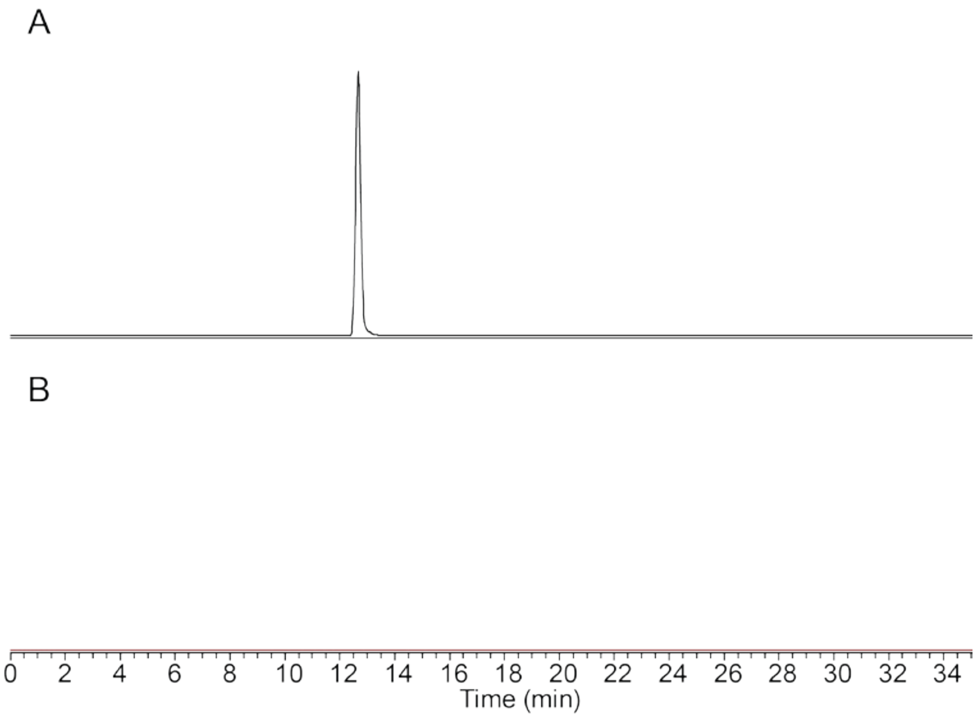

Fig. S3. Extracted (negative) ion chromatograms for *m*/z 352.0636 (glutathione-methanthiol disulfide) for *in vitro* reactions with 1-methylthio-D-xylulose 5-phosphate methylsulfurylase (A) with or (B) without glutathione as a co-substrate. Only the reaction with glutathione produced a peak with the exact mass of glutathione-methane thiol disulfide.

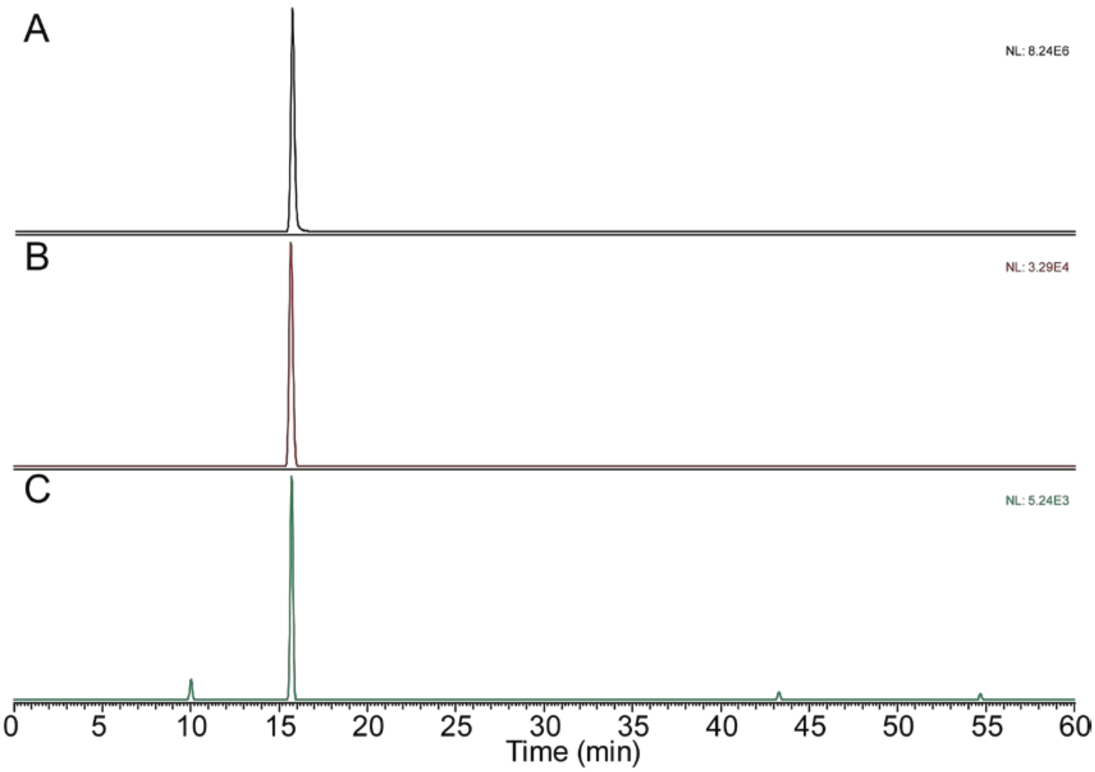

Fig. S4. Extracted (negative) ion chromatograms for *m*/z 352.0636 (glutathione-methanthiol disulfide) for MTA feeding reactions. A) Two minute *R. rubrum* MTA feeding reaction cell extract spiked with 1 µL of the in vitro reaction from Figure 5S. B) Two minute *R. rubrum* MTA feeding reaction cell extract. C) Control *R. rubrum* cell extract that had not been fed exogenous MTA. Only one peak is present in the spiked sample, thus supporting the assignment of the peak at *m/z* 352.0636 as glutathione-methanthiol disulfide in the MTA feeding reactions. The presence of glutathione-methanethiol disulfide in the unfed cell extract supports glutathione as the *in vivo* cosubstrate for 1-methylthio-D-xyulose 5-phosphate methylsulfurylase in *R. rubrum*.

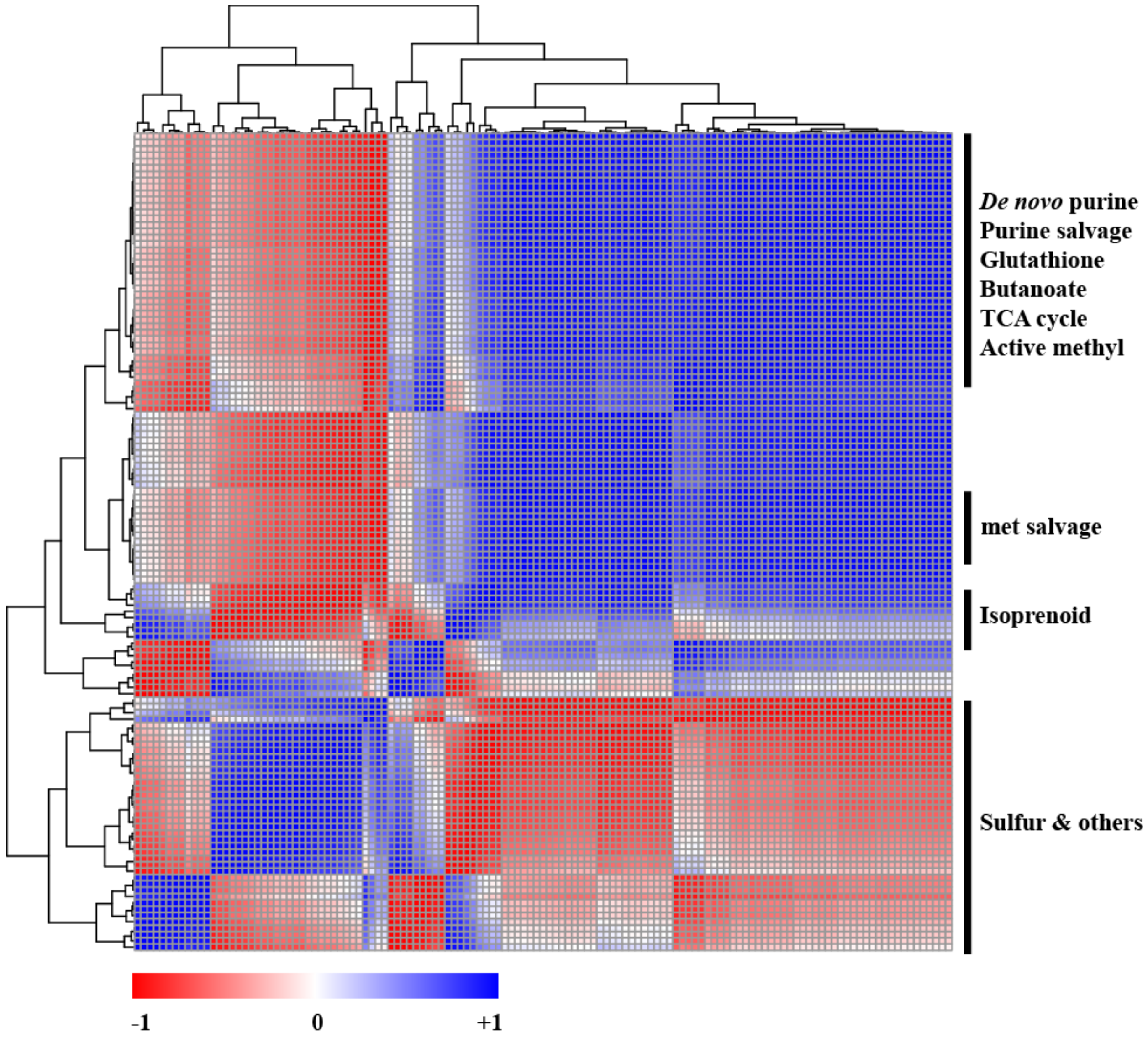

Fig. S5. Pearson correlation between metabolites in implicated pathways (*R. rubrum*). Metabolites-metabolites correlation analysis was carried out based on Pearson correlation analysis. Pearson correlation coefficients of percentage changes in metabolite abundances were clustered by agglomerative hierarchical clustering. Euclidean distance was used as a distance measure. In each pathway, there was a clear correlation between constituent metabolites. In addition, there was a strong correlation between intertwined active pathways. The non-mevalonate isoprenoid biosynthesis showed a moderate correlation, and sulfur metabolism showed an anti-correlated pattern. This is because metabolites in sulfur metabolism were down-regulated, whereas metabolites in other pathways were up-regulated.

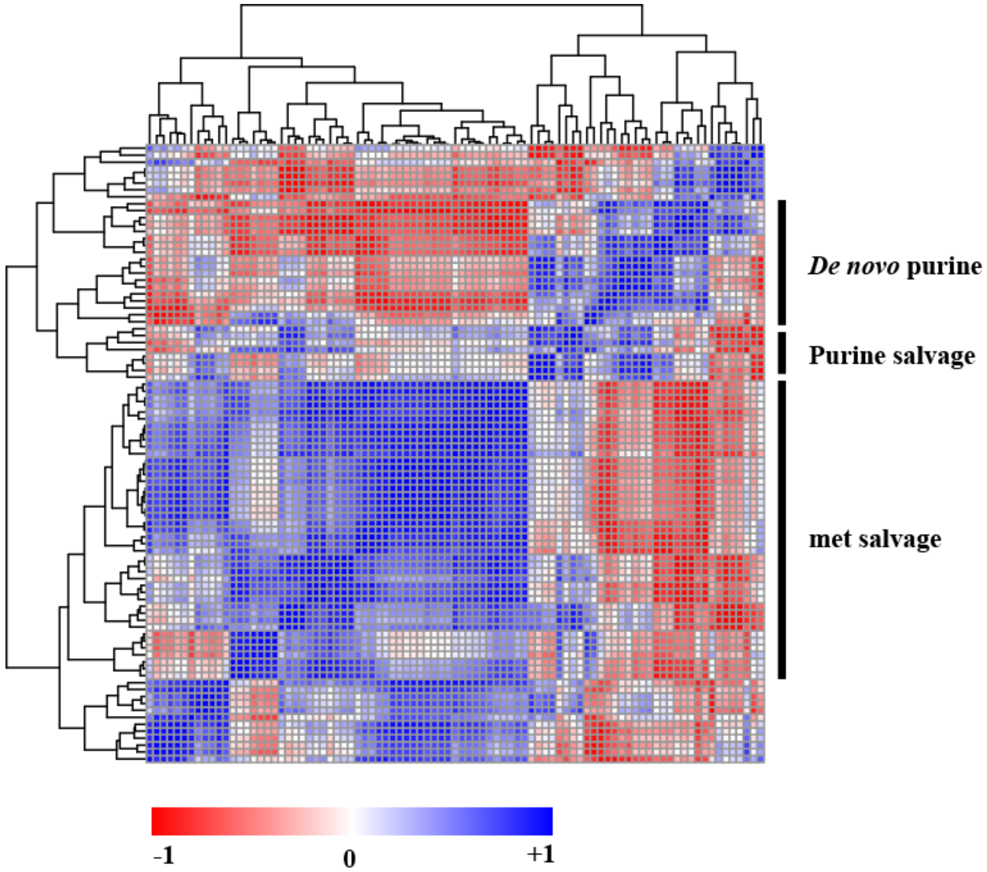

Fig. S6. Pearson correlation between metabolites in implicated pathways (*B. subtilis*). In *B. subtilis*, metabolites in each active pathway showed strong correlation, but there was a weak correlation between the subtilis-type methionine salvage pathway and the purine salvage pathway. In addition, there was an anti-correlation between *de novo* purine biosynthesis and other pathways.
